# Loss of activation by GABA in vertebrate delta ionotropic glutamate receptors

**DOI:** 10.1073/pnas.2313853121

**Published:** 2024-01-29

**Authors:** Giulio Rosano, Allan Barzasi, Timothy Lynagh

**Affiliations:** ^a^Michael Sars Centre, University of Bergen, Bergen 5008, Norway

**Keywords:** iGluR, neurotransmitter, ion channels, excitatory, GABA

## Abstract

Ionotropic glutamate receptors (iGluRs) mediate excitatory signals between cells by binding neurotransmitters and conducting cations across the cell membrane. In the mammalian brain, most of these signals are mediated by two types of iGluRs: AMPA and NMDA (i.e. iGluRs sensitive to 2-amino-3-(5-methyl-3-oxo-1,2-oxazol-4-yl)propanoic acid and N-methyl-D-aspartic acid, respectively). Delta-type iGluRs of mammals also form neurotransmitter-binding channels in the cell membrane, but in contrast, their channel is not activated by neurotransmitter binding, raising biophysical questions about iGluR activation and biological questions about the role of delta iGluRs. We therefore investigated the divergence of delta iGluRs from their iGluR cousins using molecular phylogenetics, electrophysiology, and site-directed mutagenesis. We find that delta iGluRs are found in numerous bilaterian animals (e.g., worms, starfish, and vertebrates) and are closely related to AMPA receptors, both genetically and functionally. Surprisingly, we observe that many iGluRs of the delta family are activated by the classical inhibitory neurotransmitter, γ-aminobutyric acid (GABA). Finally, we identify nine amino acid substitutions that likely gave rise to the inactivity of today’s mammalian delta iGluRs, and these mutations abolish activity when engineered into active invertebrate delta iGluRs, partly by inducing receptor desensitization. These results offer biophysical insight into iGluR activity and point to a role for GABA in excitatory signaling in invertebrates.

## Significance

The neurotransmitters glutamate and GABA activate excitatory sodium ion influx and inhibitory chloride flux across neuronal membranes by binding and activating ionotropic glutamate receptors (iGluRs) and GABA_A_ receptors, respectively, two superfamilies of ligand-gated ion channels. Curiously, mammalian receptors of the delta iGluR family mediate no sodium influx in response to neurotransmitter binding in most experimental settings, so we investigated delta iGluRs from numerous animal lineages, bioinformatically and experimentally, and found that numerous delta iGluRs are indeed ligand-gated channels. Surprisingly, most are activated by GABA, the classically inhibitory neurotransmitter. Our results identify several amino acid substitutions that occurred during evolution to make mammalian delta iGluRs inactive and reveal a potential excitatory signaling role for GABA in numerous invertebrates.

Rapid signals are conveyed between neurons of the central nervous system via chemical synapses, specialized directional interfaces between adjacent neurons (1). Most synapses in the mammalian brain are glutamatergic and excitatory, where glutamate released by the presynaptic cell binds to ionotropic glutamate receptors (iGluRs) on the postsynaptic cell (2, 3). iGluRs are tetramers assembled by four homologous subunits, each of which has an extracellular N-terminal domain (NTD), extracellular ligand-binding domain (LBD), membrane domain, and intracellular C-terminal domain (CTD). The four membrane domains together form a membrane-spanning, nonselective cation channel (3). The iGluR superfamily is broad, but two main families predominate in mammals and classical model organisms: AMPA receptors, along with close cousins kainate (KA) receptors, are rapidly activated by glutamate and depolarize cells quickly (4); NMDA receptors are slower acting, more calcium-permeable, and require the binding of glutamate and an ambient coagonist, D-Serine or glycine (5).

Additionally, there is a relatively mysterious “delta” family of iGluRs encoded by two genes, GluD1 and GluD2 in rodents and human (6–8). Like most iGluRs, delta iGluRs are expressed in excitatory synapses, their up- or downregulation leads to developmental disorders or neural malfunction, and when expressed heterologously in mammalian cells or frog oocytes, GluD1 and GluD2 subunits form homotetrameric channels (9). In stark contrast to other iGluRs, however, under numerous native and heterologous experimental conditions, no current through GluD1 or GluD2 iGluRs is activated by neurotransmitter binding (7, 10–12). This is despite two major lines of evidence that the ligands D-serine and glycine bind to the canonical iGluR LBD of GluD2 and induce local conformational change. X-ray structures of the excised GluD2 LBD show D-serine binding and LBD closure around the ligand (13), reflecting the first step of channel activation in most iGluRs (3). Second, mutant GluD2 channels carrying the “lurcher” (*Lc*) A654T substitution in the channel pore conduct current constitutively, and the binding of D-serine or glycine inactivates this current, indicative of ligand-induced conformational change in both the LBD and in the channel pore (12, 13).

The absence of typical ligand-gated currents in heterologously expressed delta iGluRs is reflected in relatively unique biological function. Intracellular signaling pathways are suggested to activate delta iGluRs based on mouse cerebellum and midbrain recordings (10, 14, 15), and a principally structural and developmental role is served by delta iGluRs in the hippocampus and cerebellum, where they interact with pre- and intrasynaptic proteins via their large extracellular NTD (16–18). NTD dynamics may in turn relate to the absence of ligand-induced gating in heterologously expressed receptors. The compaction of GluD2 extracellular domains via coexpression with synaptic proteins in densely cultured mammalian cells or via introduced cysteine-linked NTDs leads to small ligand-gated currents in heterologously expressed GluD2 iGluRs (19). Furthermore, delta iGluRs differ from AMPA and NMDA iGluRs in that cryoelectron microscopy structures of full-length GluD1 and GluD2 iGluRs show a “nonswapped” architecture, where the back-to-back NTD dimers of two adjacent subunits sit atop back-to-back LBD dimers of the same two subunits (20, 21), contrasting the “domain-swapped” architecture of other iGluRs (3). Whether nonswapped NTD structure underlies delta iGluR inactivity is doubtful, however, as plant iGluRs are nonswapped yet capable of ligand-gated currents (22).

Delta iGluR function is thus relatively mysterious. This impairs our understanding of the biophysical underpinnings of excitatory signaling, precludes the study of potential delta iGluR pharmacology, and makes biological inferences about the presence of delta iGluR genes in different animals difficult. We therefore sought to establish a molecular and functional signature of the delta iGluR family by investigating beyond the mammalian orthologues, using phylogenetics, electrophysiology, and mutagenesis. This uncovered surprisingly active ligand-gated delta iGluRs in numerous invertebrates, uncovered pharmacological similarities between delta iGluRs and their AMPA receptor cousins, and traced the inactivity of vertebrate delta iGluRs to a distinct part of the iGluR gating machinery.

## Results

### GABA-gated Channels throughout the Delta iGluR Family.

**Fig. 1.**
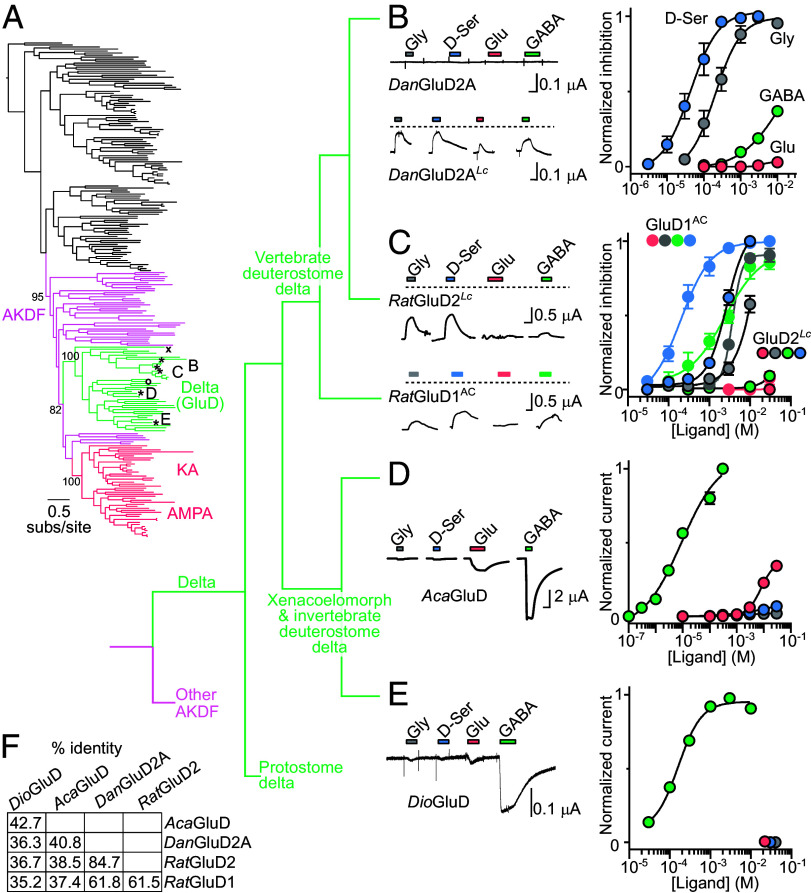
GABA-gated channels in the delta iGluR family. (*A*) Maximum likelihood phylogeny of animal iGluR genes. Selected iGluR branches are colored and labeled. *, genes characterized in panels *B*–*E*. ^X, O^, *Crassostrea gigas* GluD and *Saccoglossus kowalevskii* GluD, see *SI Appendix*, Fig. S2. SH-aLRT support for selected branches indicated. Detailed branch support and all gene names in expanded phylogeny, *SI Appendix*, Fig. S1. (*B*–*E*) Example two-electrode voltage clamp recordings of oocytes expressing indicated delta iGluR genes in response to different ligands (*Left*) and mean ± SEM (n = 4 to 6) normalized concentration-dependent responses (*Right*). Scale bars: x, 10 s; y, as indicated. (*B* and *C*) Wild-type channels were inactive; lurcher-mutant (*^Lc^*) *Dan*Glu2A and *Rat*GluD2 and A654C-mutant (^AC^) *Rat*GluD1 channels were constitutively active. The dashed line indicates zero current; mean responses reflect ligand-induced inhibition of constitutive current normalized to maximum inhibition of constitutive current. (*D* and *E*) Wild-type channels were active; mean responses reflect ligand-induced current amplitude normalized to maximum ligand-induced current amplitude. *Dan*, *Danio rerio*. *Rat*, *Rattus norvegicus*. *Aca*, *Acanthaster planci*. *Dio*, *Diopisthoporus longitibus*. (*F*) % amino acid sequence identity of selected delta iGluRs.

In questioning the divergence of relatively inactive mammalian delta iGluRs from their active, ligand-gated iGluR cousins, we sought a more definitive view of the delta iGluR family and its phylogenetic and functional relation to other iGluRs. We first generated a maximum likelihood phylogeny of iGluR genes from a broad selection of diverse animals (Fig. 1*A* and *SI Appendix*, Fig. S1). Mammalian GluD1 and GluD2 genes are found in a branch we will refer to as the delta family (green in Fig. 1*A*), whose closest relatives in terms of other previously characterized genes are AMPA/KA receptors (dark pink in Fig. 1*A*). Delta and AMPA/KA iGluRs are thus closely related, and together with several uncharacterized paraphyletic relatives make up the AMPA/KA/delta/phi (AKDF) branch that was proposed by others (23, 24). The delta iGluR family comprises genes only from animals of the bilaterian lineage, i.e., xenacoelomorphs, a distinct group of simple marine worms lacking a circulatory system (25); protostomes such as molluscs; and deuterostomes such as vertebrates, hemichordates (e.g., acorn worms), and echinoderms (e.g., starfish, *SI Appendix*, Fig. S1). According to our maximum likelihood phylogeny, protostome delta iGluRs are the earliest branching within the delta family (^X^ in Fig. 1*A*), consistent with a previously published Bayesian phylogeny (23).

Apart from inactive wild-type (WT) and constitutively active, D-serine-inhibited, mutant mammalian delta iGluRs, the functional signature of the delta iGluR family is unknown. We therefore expressed putative delta iGluRs from diverse bilaterian animals in frog oocytes and characterized their function with two-electrode voltage clamp. Genes included “*Dio*GluD” from the acoel *Diopisthoporus longitubus*, (a xenacoelomorph); “*Cra*GluD” from the oyster *Crassostrea gigas* (a protostome); “*Aca*GluD” from the crown-of-thorns-starfish *Acanthaster planci* (an echinoderm) and “*Sac*GluD” from the acorn worm *Saccoglossus kowalevskii* (a hemichordate; both invertebrate deuterostomes); and “*Dan*GluD2A” from the zebrafish *Danio rerio* (a vertebrate deuterostome). Similar to *Rat*GluD1 and *Rat*GluD2, WT *Dan*GluD2A receptors showed no response to the four ligands tested, but lurcher-mutant (*Lc*) *Dan*GluD2A*^Lc^* receptors showed constitutive currents that were inactivated by D-serine and glycine (Fig. 1*B*). However, in contrast to *Rat*GluD2*^Lc^*, which were only sensitive to glycine and D-serine, *Dan*GluD2A*^Lc^* receptors and *Rat*GluD1^AC^ [the *Lc*-like A654C mutation (26)] were also sensitive to GABA, which caused 58 ± 8% (n = 4) or 86 ± 5% (n = 10) inactivation relative to D-serine at these two receptors (Fig. 1 *B* and *C*). This shows that numerous vertebrate delta iGluRs are inactive, but suggests that some of them bind the classical inhibitory neurotransmitter GABA, as indicated by a recent study of *Rat*GluD1 (27).

In stark contrast to WT delta iGluRs of vertebrate deuterostomes, WT delta iGluRs of other bilaterians showed robust ligand-gated currents, and remarkably the most effective agonist was GABA (Fig. 1 *D* and *E*). At invertebrate deuterostome (starfish) *Aca*GluD, the GABA EC_50_ of 13 ± 3 μM (n = 5) was much lower than that of glutamate (7.8 ± 3 mM, n = 3). Fellow invertebrate deuterostome (acorn worm) *Sac*GluD showed smaller currents, making potency difficult to measure, although GABA was more potent than other potential ligands (*SI Appendix*, Fig. S2*A*). This was more evident in lurcher-mutant *Sac*GluD*^Lc^*, which showed relatively small constitutive current and inward GABA-gated currents of very high potency (EC_50_ = 84 ± 14 nM, n = 4, *SI Appendix*, Fig. S2 *B*–*D*). At xenacoelmorph (acoel) *Dio*GluD iGluRs, GABA was in fact the only ligand that elicited currents, with an EC_50_ of 180 ± 20 μM (Fig. 1*E*). Finally, we tried measuring the activity of the earliest branching delta iGluR in our tree, protostome (oyster) *Cra*GluD (^X^ in Fig. 1*A*). Unfortunately, we could not establish the function of this receptor, as we detected no ligand-gated currents in oocytes injected with WT or *Lc* mutant *Cra*GluD mRNA, probably due to low surface expression, as assayed by oocyte immunolabeling (*SI Appendix*, Fig. S2 *E* and *F*).

Inferring the putative functional properties of the ancestral receptor at the base of the delta branch is difficult without functional data on early-branching protostome delta iGluRs. However, considering the presence of a) GABA sensitivity in both xenacoelomorph, deuterostome invertebrate, and certain deuterostome vertebrate delta iGluRs, b) the absence of ligand-gated currents in WT vertebrate delta iGluRs, and c) the fact that AMPA/KA iGluR cousins are glutamate-gated channels, we suggest the following. When the first delta iGluR diverged from its AKDF ancestor, it was an active ligand-gated channel that quickly evolved selectivity for the neurotransmitter GABA. After chordates diverged from other invertebrate deuterostomes, ligand-induced channel gating was lost in delta iGluRs of the chordate or subsequent vertebrate lineage. And based on the presence of both GABA and glycine/D-serine sensitivity in vertebrate GluD1 and GluD2 receptors, we tentatively conclude that ligand selectivity changed from GABA to glycine and D-serine in early vertebrates or other chordates.

### Computational Analysis of Ligand Binding.

**Fig. 2.**
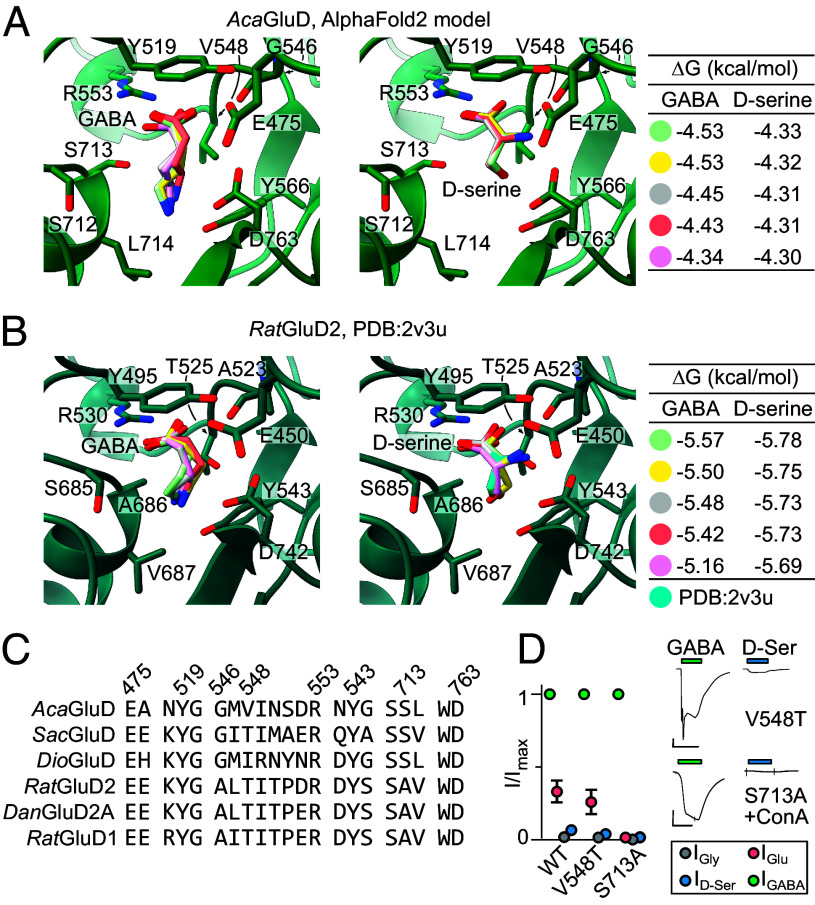
Computational ligand docking. (*A* and *B*) Five most energetically favorable binding poses for GABA and D-serine at *Aca*GluD model (*A*) and *Rat*GluD2 X-ray structure [PDB:2v3u (13)] (*B*). (*C*) Amino acid sequence alignment of selected LBD segments (*Aca*GluD numbering). (*D*) Example recording and mean (± SEM, n = 4) normalized responses of indicated mutant *Aca*GluD receptors to different ligands. Oocytes expressing S713A receptors only responded after concanavalin A treatment (“ConA”, 10 μM).

One potential explanation for the evolutionary scenario described above would involve substitutions in the ligand-binding residues of the vertebrate delta iGluR LBD, enabling selectivity for α-amino acid D-serine (and glycine) instead of γ-amino acid GABA. We investigated this by computationally docking GABA and D-serine to a starfish *Aca*GluD AlphaFold structural model and the *Rat*GluD2 X-ray structure (13). With an upper lobe–lower lobe separation similar to that of D-serine-bound *Rat*GluD2 (ref. 13 and *SI Appendix*, Fig. S3 *A* and *D*), our model likely represents an active ligand-bound conformation. As expected, based on experimental evidence for *some* sensitivity to GABA and D-serine (Fig. 1 *C* and *D*), both ligands docked to both receptors in the canonical binding site, but with more favorable energies for GABA than D-serine at *Aca*GluD and the converse at *Rat*GluD2 (Fig. 2 *A* and *B* and *SI Appendix*, Fig. S3).

Aligning the ligand-binding residues shows only minor differences between GABA-selective *Aca*GluD and D-serine- (and glycine-) selective *Rat*GluD2 (Fig. 2*C*). Indeed, the major bonding partner of the GABA γ-amine and D-serine α-amine in both receptors is the carboxylate side chain of the conserved lower lobe D763/D742 residue (Fig. 2 *A*–*C*). Similarly, although the upper lobe E475/E450 carboxylate residue was recently shown to contribute to GABA potency in *Rat*GluD1 (27), it is conserved among GABA-selective invertebrate and D-serine-selective vertebrate delta iGluRs (Fig. 2 *A*–*C*), and thus does not determine ligand selectivity.

We did notice two differences between the receptors, however. In the upper lobe, hydrophobic V548 of *Aca*GluD cannot form a polar interaction with the α-amine of D-serine that is formed by the equivalent but polar T525 of *Rat*GluD2 (Fig. 2 *A*–*C*). And in the lower lobe, invertebrate receptors have a polar S713 side chain where vertebrate receptors have a small, nonpolar A686 side chain (Fig. 2 *A*–*C*). However, when we tested the ligand selectivity profile of mutant V548T and S713A *Aca*GluD receptors, we saw no increase in D-serine activity relative to GABA (Fig. 2*D*), suggesting that these differences do not determine ligand selectivity. This is reflected in the fact that invertebrate *Sac*GluD, which has the upper lobe threonine residue like *Rat*GluD2, shows high GABA selectivity (Fig. 2*C* and *SI Appendix*, Fig. S2*D*). Taken together, these computational and functional data show that most extant delta iGluRs either show GABA selectivity or retain some GABA sensitivity and that ligand-binding residues alone do not determine ligand selectivity in delta iGluRs.

### Pharmacological and Biophysical Properties of Delta iGluRs Reflect their Close Relationship to AMPA/KA Receptors.

Although phylogenetic relationships suggest that delta iGluRs are closely related to AMPA/KA receptors, previous pharmacological studies have emphasized similarities between delta iGluRs and NDMA receptors, such as glycine and D-serine binding, and channel block by pentamidine (19, 28). We therefore sought a more extensive view of delta iGluR function, incorporating channel pore properties, competitive antagonist pharmacology, and modulation by extracellular calcium ions (Ca^2+^), utilizing the crown-of-thorns starfish *Aca*GluD receptor because of its large, tractable ligand-gated currents in oocytes.

**Fig. 3.**
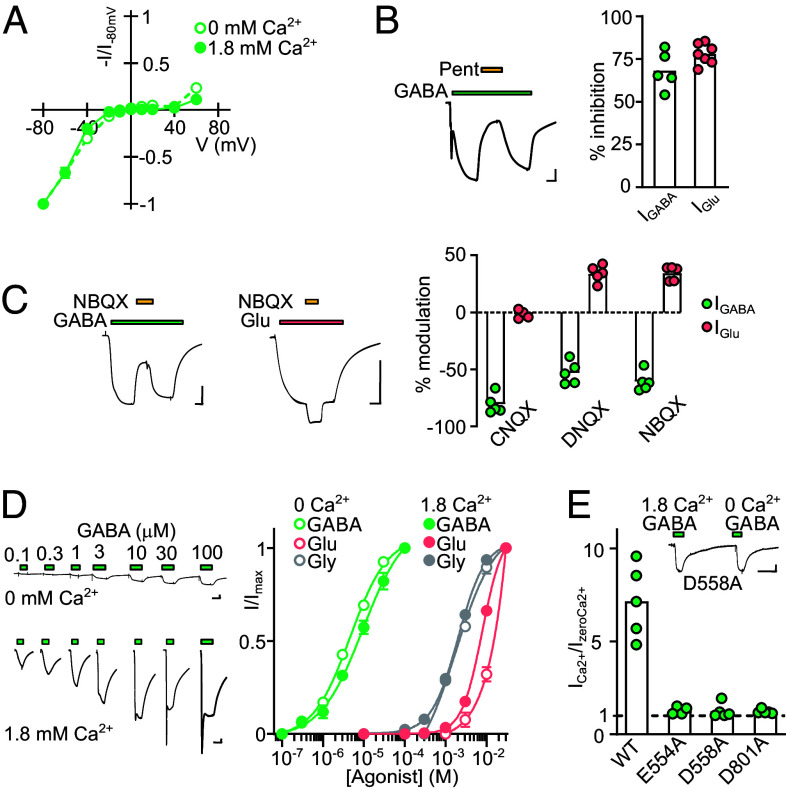
Pore properties and pharmacology of *Aca*GluD receptors. (*A*) Normalized current (I/I_−80 mV_) -voltage relationship of GABA-gated currents through *Aca*GluD-expressing oocytes. Data points mean ± SEM (n = 4), joined by straight lines. (*B* and *C*) Example recordings (scale bars x, 5 s; y, 1 μA) and summary data (columns, mean; circles, individual data points) of modulation of GABA- and glutamate-gated current by indicated drugs at *Aca*GluD-expressing oocytes. (*D*) Example responses to increasing concentrations of GABA in the absence or presence of extracellular Ca^2+^ (*Left*) and normalized (to maximum current in respective condition, “I/I_max_”) responses to different agonists (*Right*, mean ± SEM, n = 5 to 7). (*E*) Fold enhancement (“I_Ca2+_/I_zeroCa2+_”) of GABA-gated current by 1.8 mM Ca^2+^ at oocytes expressing WT or indicated mutant *Aca*GluD. *Inset*: example recording from oocyte expressing D558A mutant *Aca*GluD, scale bars in *D* and *E*: x, 10 s; y, 0.15 μA.

To establish the channel pore properties of *Aca*GluD receptors we measured current-voltage (IV) relationships and channel block by pentamidine. The IV relationship at *Aca*GluD iGluRs is inwardly rectifying, with small outward currents only appearing at potentials more positive than 50 mV, both in the absence and presence of divalent cations (Fig. 3*A*). This suggests that *Aca*GluD receptors are blocked by intracellular polyamines and not by extracellular Mg^2+^ ions, which is qualitatively similar to AMPA receptors (29) and chimeric receptors carrying KA receptor LBDs and *Rat*GluD2 channel domains (30). Running voltage ramps from −80 to 60 mV during GABA-gated currents in regular extracellular solution yielded a reversal potential of −16 ± 2 mV (n = 4), as expected for a nonselective cation-permeable channel in these conditions (31). This indicates that *Aca*GluD is a mixed cation channel like most iGluRs (3) and thus an excitatory GABA receptor.

Pentamidine is a diarylamidine compound previously shown to block NMDA receptors in the low micromolar range and constitutively active mutant *Rat*GluD2*^Lc^* channels in the high micromolar range (28, 32). We observed that pentamidine (100 µM) blocked 68 ± 5% (n = 5) of the GABA-gated current and 78 ± 2% (n = 7) of the glutamate-gated current through *Aca*GluD iGluRs (Fig. 3*B*). As the pentamidine sensitivity of AMPA receptors has been explored relatively little, we tested this ourselves and observed that pentamidine (100 µM) elicited 37 ± 3% (n = 5) inhibition of glutamate-gated current through *Rat*GluA2 AMPA receptors (*SI Appendix*, Fig. S4*A*). Combined with earlier studies, our results show that AMPA receptors and delta iGluRs share moderate pentamidine sensitivity, in contrast to NMDA receptors, which have higher pentamidine sensitivity.

Applied alone, the classical AMPA/KA receptor competitive antagonists CNQX, DNQX, and NBQX (100 μM) elicited no currents at *Aca*GluD iGluRs, but when coapplied with GABA (~EC_30_ GABA concentration) inhibited 81 ± 4%, 53 ± 3%, and 61 ± 3% of the GABA-gated current, respectively (Fig. 3*C*, each n = 5). In contrast, the NMDA receptor glycine site antagonists 5,7-dichlorokynurenic acid (33) and CGP-78608 (34) (both 100 μM) inhibited only 11 ± 5% and 9 ± 3% of the GABA-gated currents, respectively (*SI Appendix*, Fig. S4*B*). Curiously, glutamate-gated currents were *enhanced* 34 ± 3% by DNQX and NBQX, whereas CNQX had no effect (Fig. 3*C*, each n = 5). These quinoxalinedione compounds typically inhibit heterologously expressed AMPA/KA receptors, although CNQX and DNQX act as partial agonists at AMPA receptors coexpressed with TARP-type auxiliary subunits (35). Thus, at both delta and AMPA/KA receptors, certain quinoxalinediones exert complex effects, depending on the specific agonist or oligomeric complex. We observed no effect of CNQX, DNQX, or NBQX on ooyctes expressing WT *Rat*GluD2 (n = 4), but constitutive currents through *Rat*GluD2*^Lc^* were inhibited 10 ± 0.2%, 11 ± 0.2%, and 9 ± 0.2% by CNQX, DNQX, and NBQX, respectively (each n = 4, *SI Appendix*, Fig. S4*C*), consistent with the moderate inhibition of *Rat*GluD2*^Lc^* currents previously reported for CNQX and another, different quinoxalinedione (36).

Further assessing the functional signature of delta iGluRs, we tested *Aca*GluD for sensitivity to extracellular Ca^2+^, which was previously shown to enhance the constitutive activity of mutant *Rat*GluD2*^Lc^* channels and decrease the potency with which D-serine inhibits constitutive activity (37). We observed that extracellular Ca^2+^ enhanced *Aca*GluD substantially. In nominally Ca^2+^-free solutions, maximum GABA-, glutamate-, and glycine-gated current amplitudes were 0.42 ± 0.13 µA (n = 4), 0.28 ± 0.02 µA (n = 5), and 0.08 ± 0.01 μA (n = 10), and these were increased to 6.0 ± 1.0 µA (n = 5), 5.8 ± 0.6 µA (n = 4), and 0.33 ± 0.01 μA (n = 4) in the presence of 1.8 mM Ca^2+^ (Fig. 3 *D* and *E*). Enhancement affected maximum current amplitude more than agonist potency, with no more than threefold altered agonist EC_50_ values in 1.8 mM Ca^2+^ (Fig. 3*D*; GABA EC_50_ 5 ± 0.6 µM in 0 Ca^2+^ and 13 ± 3 µM in 1.8 mM Ca^2+^, n = 5; glycine EC_50_ 2 ± 0.1 mM and 2 ± 0.1 mM, n = 4; glutamate EC_50_ ~30 mM and ~10 mM, n = 4). We tested whether *Aca*GluD enhancement by Ca^2+^ is determined by carboxylate side chains on the rear of the LBD as was shown for *Rat*GluD^Lc^ receptors (37). E554A, D558A, and D801A mutant *Aca*GluD iGluRs each showed no enhancement by Ca^2+^ (Fig. 3*E* and *SI Appendix*, Fig. S4*D*), indicating that equivalent, conserved positions determine Ca^2+^ sensitivity in starfish and rat delta iGluRs (*SI Appendix*, Fig. S5). Thus, enhancement by extracellular Ca^2+^ is conserved in diverse delta iGluRs, and mixed cation permeability, inward rectification, quinoxolinedione sensitivity, and sensitivity to high micromolar pentamidine are all conserved in delta iGluRs and their AMPA receptor cousins.

### The NTD Does not Determine the Ligand-gated Activity of Starfish Delta iGluRs.

Our data suggest that after diverging from other AKDF iGluRs, early delta iGluRs were active, homotetrameric ligand-gated channels, even in the absence of accessory synaptic proteins. And while this ligand-gated activity is conserved in several extant delta iGluRs, delta iGluRs in the lineage to vertebrates lost ligand-gated channel activity. We questioned the molecular basis for this loss, hoping to establish a molecular blueprint for the mysterious function of mammalian delta iGluRs.

**Fig. 4.**
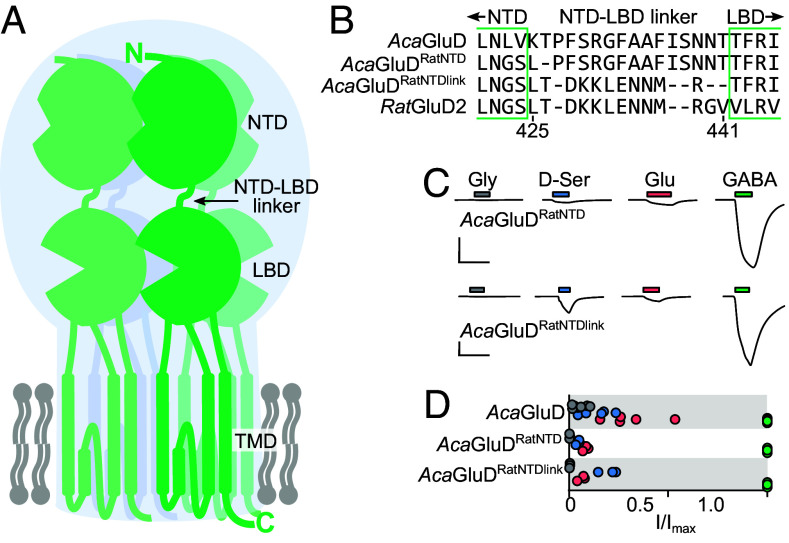
*Rat*GluD2 NTD does not abolish the activity of starfish *Aca*GluD iGluRs. (*A*) Cartoon illustrating structural domains of typical iGluR tetramer, one subunit highlighted, showing amino-terminal domain (NTD), ligand-binding domain (LBD), and transmembrane channel domain (TMD). Cell membrane in gray. (*B*) Amino acid sequence alignment of linker region, illustrating chimera design. *Numbering*, *Rat*GluD2. (*C*) Ligand-gated currents in oocytes expressing mutant *Aca*GluD receptors containing the NTD from *Rat*GluD2 (*Aca*GluD^RatNTD^) or the NTD and NTD-LBD linker from *Rat*GluD2 (*Aca*GluD^RatNTDlink^). Scale bars: x, 10 s; y, 2 µA. (*D*) Summarized data from experiments in *C*.

In most animal iGluR subunits, the large clamshell-shaped NTD contributes greatly to tetramer assembly but relatively moderately to ligand-gated channel activity (3). However, NTDs are indirectly implicated in the ligand-gated (in)activity of mouse/human delta iGluRs, as the experimental separation of NTDs from each other, or from the LBDs below, impairs channel function (17, 19). We therefore questioned whether substantial divergence in the NTDs—19% amino acid sequence identity between *Aca*GluD and *Rat*GluD2 NTDs *cf* 38% identity between *Aca*GluD and *Rat*GluD2 LBD and channel domains—determines the absence of ligand-gated currents in vertebrate delta iGluRs. To answer this, we measured ligand-gated currents in two chimeric *Aca*GluD constructs in which the NTD was replaced with that of *Rat*GluD2: one retaining the NTD-LBD linker of *Aca*GluD, “*Aca*GluD*^Rat^*^NTD^”; and the other with the NTD-LBD linker of *Rat*GluD2, “*Aca*GluD*^Rat^*^NTDlink^” (Fig. 4 *A* and *B* and *SI Appendix*, Supporting text). Neither chimeric receptor showed drastic differences from WT *Aca*GluD, with each showing large GABA-gated currents (Fig. 4 *C* and *D*). However, we did notice a small increase in the relative efficacy of D-serine at *Aca*GluD*^Rat^*^NTDlink^ (20 ± 3%, n = 3) compared to WT and *Aca*GluD*^Rat^*^NTD^ (12 ± 3%, n = 7, and 5 ± 1%, n = 3, respectively). While confirming that the NTDs and their link to the LBD make some sort of contribution to delta iGluR function (17), these results show that great divergence in the NTDs is unlikely to have determined the stark loss of function in vertebrate delta iGluRs relative to other deuterostome delta iGluRs.

### Vertebrate-like Mutations in the Lower Lobe of the LBD Abolish the Activity of Starfish Delta iGluRs.

These results suggest that the relative inactivity of vertebrate delta iGluRs such as *Rat*GluD2 derives from amino acid sequence divergence in the remainder of the receptor, i.e., the LBD, transmembrane channel domain (TMD), and the intracellular CTD. We therefore aligned amino acid sequences of delta iGluR subunits, and from ~410 positions comprising the LBD and TMD, we identified 41 at which the amino acid residue shares biophysical properties in *Aca*GluD, *Sac*GluD, and *Dio*GluD and differs in inactive *Dan*GluD2A, *Rat*GluD1, and *Rat*GluD2 (*SI Appendix*, Fig. S5). As the long CTD is implicated little in channel gating and more in expression and trafficking of iGluRs (3), we excluded it from this analysis. Taking *Aca*GluD again as our active experimental model, we substituted each of these 41 amino acid residues with the equivalent amino acid from inactive *Rat*GluD2, generating 39 mutant *Aca*GluD receptors, which we tested for responses to GABA, glutamate, glycine, and D-serine. (Two of the mutants incorporated substitutions of two adjacent residues for efficiency.)

**Fig. 5.**
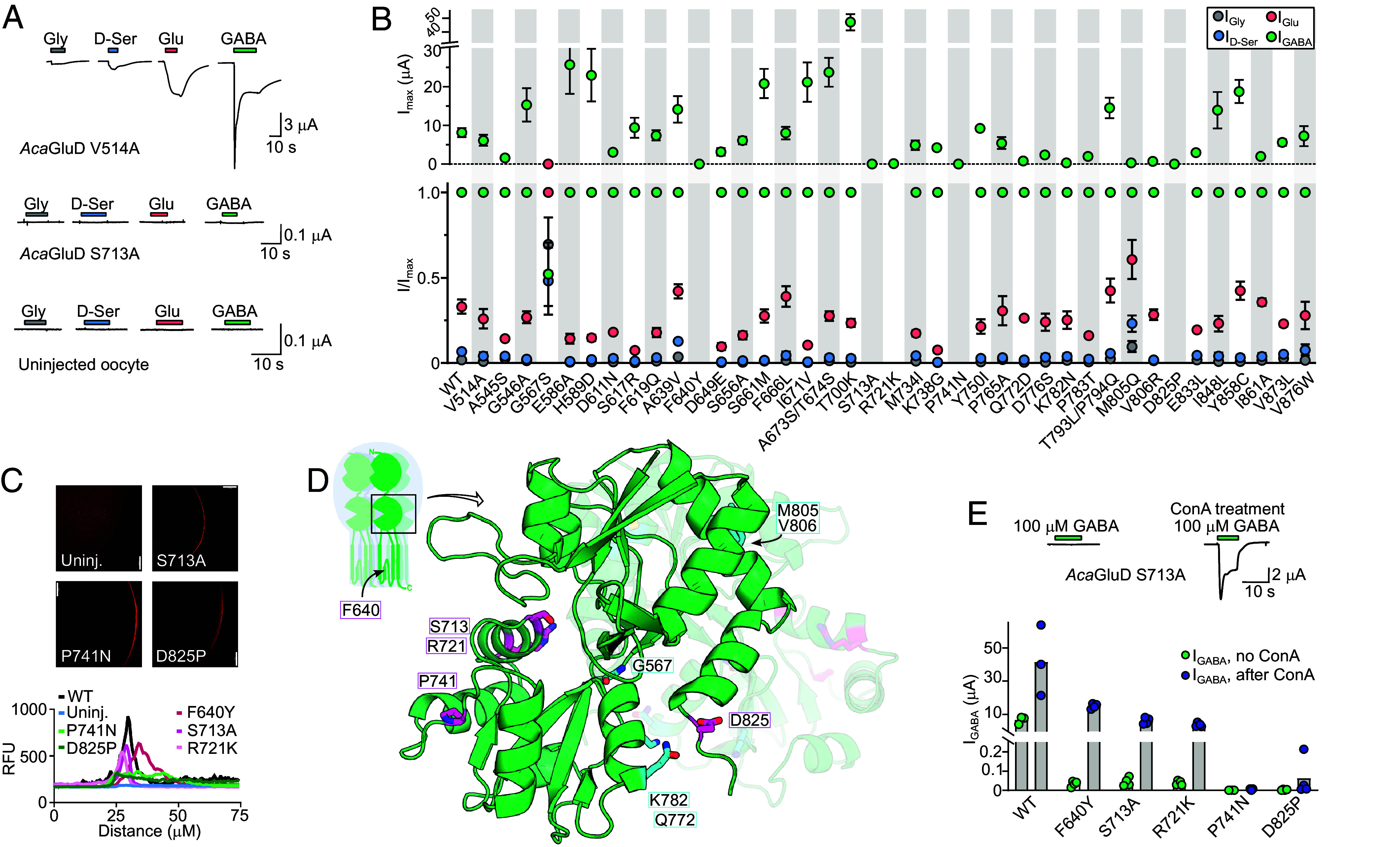
*Rat*GluD2-like substitutions impair the ligand-gated activity of *Aca*GluD receptors. (*A*) Ligand-gated currents in oocytes expressing indicated mutant *Aca*GluD receptors or uninjected oocytes. (*B*) Maximum current amplitude (“I_max_”, *Upper*) and relative ligand-gated current amplitude (“I/I_max_”, *Lower*) and at wild-type (WT) and mutant *Aca*GluD receptors (mean ± SEM, n = 3 to 5). (*C*) Immunolabeling c-Myc tag in *Aca*GluD C-terminal. *Upper*, example micropgraphs. *Lower*, Random fluorescence units (RFU) plotted against relative radial distance, peaking at the oocyte surface. White scale bars, 100 μm. (*D*) AlphaFold model of two adjacent *Aca*GluD LBDs (rear LBD faded). Selected amino acid residues colored and shown as sticks. *Magenta*, drastic loss of function; *cyan*, moderate loss of function. Inset cartoon shows LBDs within full-length receptor and approximate position of F640 residue (*SI Appendix*, Fig. S6*A*). (*E*) *Upper*, example, and *Lower*, summarized current responses to 30 mM GABA (“I_GABA_”) in WT or mutant *Aca*GluD-expressing oocytes before or after treatment with concanavalin A (“ConA”, 10 μM).

Thirty-five of the 41 substitutions had little or no effect on *Aca*GluD activity, with the respective mutants showing large responses to GABA and smaller responses to the other agonists (Fig. 5 *A* and *B*). In contrast, six substitutions substantially altered function, with the respective mutants showing either no currents greater than uninjected oocytes (F640Y, S713A, R721K, P741N, and D825P) or a substantial change in relative agonist efficacies combined with decreased current amplitude (G567S; Fig. 5 *A* and *B*). The loss of currents via F640Y, S713A, and R721K substitutions derives primarily from altered channel function and not decreased oocyte surface expression, as the latter was similar to WT, whereas for P741N and D825P, surface expression was evident but greatly reduced compared to WT (Fig. 5*C*). An additional four mutants, Q772D, K782N, M805Q, and V806R, showed milder but noticeable effects, decreasing maximum GABA-gated current amplitude to less than 1 μA (cf. 8.2 ± 1.1 μA at WT, n = 4) and retaining the typical agonist selectivity profile (Fig. 5*B*).

Questioning how these 10 residues contribute to receptor function, we considered their putative location in the receptor tertiary structure. For the LBD, we used the AlphaFold structural model, and for the TMD, we examined the position of homologous residues in published rat delta and AMPA receptor structures (13, 20, 21, 38). From the 10 crucial *Aca*GluD delta iGluR residues, F640 is in the second helix of the TMD, abutting the channel pore (*SI Appendix*, Fig. S6*A*), whereas the other nine residues are all in the LBD, remarkably with seven specifically in the lower lobe of the LBD clamshell (Fig. 5*D*). Ligand-induced activation of iGluRs involves the upward/outward movement of the lower lobe toward the upper lobe, closing the clamshell around the ligand and pulling the pore-lining third TMD helix outward to open the channel (38, 39). Eight of the nine residues are thus in a crucial part of the receptor, close to the putative ligand binding site in the cleft of the LBD clamshell but probably located and/or oriented away from the putative ligand (Fig. 5*D* and *SI Appendix*, Fig. S6*B*). Indeed, G567, R721, D825, and K782 of the lower lobe and M805 and V806 of the upper lobe are all on the “rear” of the LBD, interfacing with the rear of an adjacent LBD within one of two LBD dimers in the homotetramer (Fig. 5*D*). P741 is in the outer lip of the lower lobe, and S713 is more central (Fig. 5*D*), although its hydroxyl moiety is unlikely to interact directly with GABA or other ligands (Fig. 2).

### Vertebrate-like Mutations in the Lower Lobe of the LBD Induce an Inactive Channel State.

In AMPA/KA iGluRs, interactions between interfacing LBDs control entry into or recovery from agonist-induced desensitization (40–43). Given the relation of delta and AMPA/KA iGluRs, and the position of loss-of-function *Aca*GluD substitutions S713A, R721K, P741N, and D825P in or near the interface of LBDs, we hypothesized that the loss-of-function may derive from altered LBD dynamics and increased desensitization compared to WT *Aca*GluD. We therefore tested responses of mutant *Aca*GluD iGluRs after treatment with concanavalin A, a plant lectin that prevents desensitization and thus uncovers otherwise small responses in some iGluRs (44, 45). Concanavalin A treatment caused a remarkable (>>100-fold) increase in F640Y, S713A, and R721K *Aca*GluD iGluR activity, with GABA-gated currents now resembling those through WT channels (Fig. 5*E*). The same restoration of function was not observed with P741N and D825P mutants, although a relatively small fourfold increase in current amplitude was observed in D825P channels, similar to WT channels (Fig. 5*E*). Thus, active delta iGluRs are rendered inactive by three vertebrate delta iGluR-like mutations that bias the receptor toward desensitization or another inactive state, and two additional vertebrate delta iGluR-like mutations primarily reduce surface expression of active delta iGluRs.

Considering that such desensitization or inactivation may underlie the absence of currents in WT vertebrate delta iGluRs, we tested the effects of D-serine and glycine on *Rat*GluD1 and *Rat*GluD2 after concanavalin A treatment, but we observed no difference to inactive, untreated receptors (*SI Appendix*, Fig. S4*E*). As *Rat*GluD1 and *Rat*GluD2 contain only three predicted N-linked glycosylation sites per subunit compared to eight in *Aca*GluD (seven in the S713A mutant), however, it could be that concanavalin A is incapable of modulating the vertebrate receptors to rescue them from such a state, precluding conclusions along these lines.

### Starfish-like Mutations Partly Reawaken Inactive Rat Delta iGluRs.

**Fig. 6.**
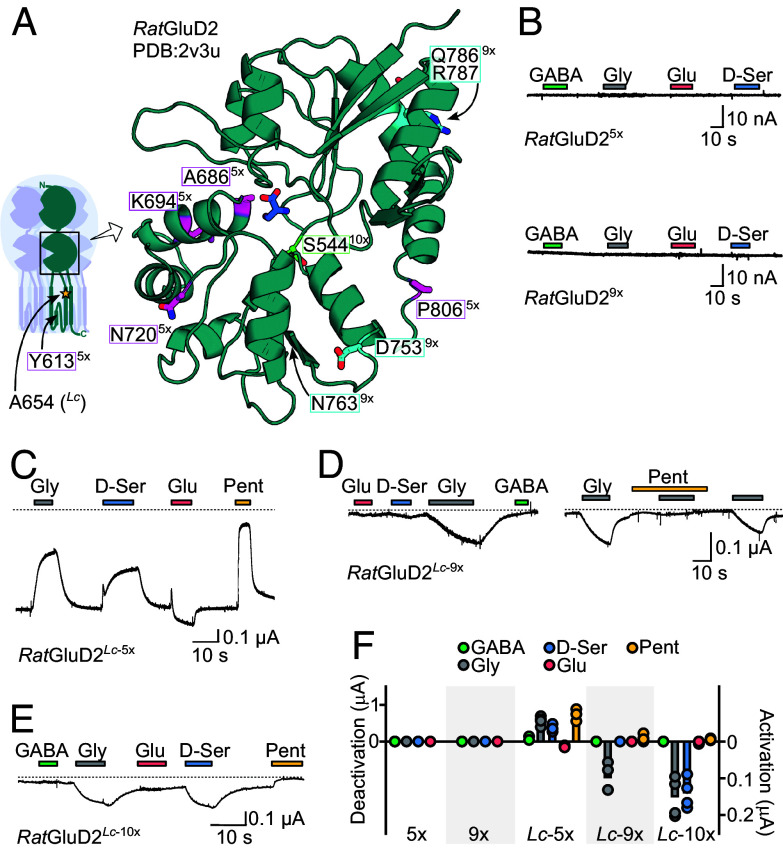
*Aca*GluD-like mutations alter *Rat*GluD2 receptor function. (*A*) D-serine bound *Rat*GluD2 from X-ray structure PDB:2v3u (13). Selected amino acid residues are indicated, colored, shown as sticks, and labeled 5×, 9×, 10×, and/or *Lc* according to the multiple mutants they were incorporated into. 10× mutant included 10×, 9×, and 5× positions. 9× included 9× and 5×. 5× included only 5×. The *Inset* cartoon shows LBD within full-length receptor and approximate position of two other residues. (*B*–*E*) Example current responses to different neurotransmitter ligands (30 mM) and to pore blocker pentamidine (Pent, 100 μM) in oocytes expressing indicated *Rat*GluD2 mutants. The dashed line indicates zero current baseline. (*F*) Individual (dots) and mean (columns) responses to ligands in oocytes expressing indicated *Rat*GluD2 mutants (n = 4 to 5).

If the 10 mutations discussed above drove the loss of function in vertebrate delta iGluRs, one might expect that the latter could be “reawakened” via active invertebrate delta iGluR-like mutations here. We therefore engineered mutant *Rat*GluD2 iGluRs to contain at these positions the equivalent residues from starfish delta iGluRs. These mutants were *Rat*GluD2^5x^, containing Y613F, A686S, K694R, N720P, and P806D substitutions (pink in Fig. 6*A*), and *Rat*GluD2^9x^, additionally containing N763K, D753Q, Q786M, and R787V (cyan in Fig. 6*A*). We also generated A654T *Lc*-mutant versions, *Rat*GluD2*^Lc^*^-5x^ and *Rat*GluD2*^Lc^*^-9x^, hypothesizing that constitutively active *Lc*-versions could offer tangible insight on ligand sensitivity in case the former mutants remained inactive.

*Rat*GluD2^5x^ and *Rat*GluD2^9x^ mutants showed no responses to glycine, D-serine, GABA, or glutamate (Fig. 6*B*), indicating that these nine mutations alone are not capable of reawakening ligand-gated currents in vertebrate delta iGluRs. On the artificial *Lc*-mutant background, however, tangible effects of the starfish GluD-like mutations were observed. Whereas *Rat*GluD2*^Lc^*^-5x^ behaved much like the typical *Lc*-mutant *Rat*GluD2*^Lc^*, *Rat*GluD2*^Lc^*^-9x^ receptors were inactive at rest and conducted inward currents in response to glycine binding (Fig. 6 *C* and *D*). These glycine-gated currents were inhibited by the pore blocker pentamidine (Fig. 6*D*). Finally, we generated *Rat*GluD2*^Lc^*^-10x^, additionally containing S644G (light green in Fig. 6*A*), the converse of the G567S substitution that altered ligand selectivity in starfish *Aca*GluD. Ligand selectivity was slightly altered by the addition of the S644G mutation, as *Rat*GluD2^10x^ receptors showed inward currents in response to both glycine and D-serine (Fig. 6 *E* and *F*). Thus, the reintroduction of key residues from active delta iGluRs into inactive vertebrate delta iGluRs is not enough to reawaken the latter, but on an artificial *Lc*-mutant background, it awakens the gating machinery, leading to *Rat*GluD2 channels that are inactive at rest and activated by glycine and D-serine binding.

## Discussion

The relative inactivity of mammalian delta iGluRs compared to other iGluRs has intrigued biophysicists, impaired pharmacological developments, and confounded our inferences from genome sequencing data. We therefore investigated the evolution and biophysical properties of the delta iGluR family, uncovering GABA-gated delta iGluRs in numerous invertebrates, dissecting their relationships with other iGluRs, and identifying a putative molecular basis for the loss of activity in vertebrate delta iGluRs.

### Divergence of Delta Receptors from Other iGluRs.

Our phylogenetic analysis finds delta iGluR genes only in bilaterians, consistent with a previous, detailed phylogenetic study (23). But as our study also included xenacoelomorphs, a group of bilaterians that likely branched before the divergence of various nephrozoans (i.e., protostomes and deuterostomes) (46), we now suggest that delta iGluRs emerged from the duplication of an AKDF gene in early bilaterian animals, shortly after they split from cnidarians (e.g., sea anemones and jellyfish).

Delta iGluRs form a family within the AKDF branch of the iGluR superfamily, a branch that includes the AMPA/KA family of fast-activating and deactivating glutamate-gated channels and numerous other uncharacterized genes. We cannot infer the functional nature of the very first delta iGluR, because we were unable to measure the activity of early branching delta iGluRs found in protostomes. Nor do we know the functional nature of the gene from which the first delta iGluR emerged, as much of the sister group to delta iGluRs, the combined [(AMPA/KA)+(Phi)+(uncharacterized AKDF)] branch, is uncharacterized, and branch support toward the base of the AKDF branch is relatively weak (*SI Appendix*, Fig. S1 and ref. 23). However, the presence of GABA sensitivity in xenacoelomorph, invertebrate deuterostome, and even certain vertebrate delta iGluRs, suggests that early delta iGluRs were GABA-sensitive. And the ligand-gated channel activity of numerous delta iGluRs, together with that of AMPA/KA receptors, suggests that mutations in delta iGluR genes of either chordates or vertebrates led to the inactivity of extant vertebrate delta iGluRs.

### Biophysical Mechanisms.

We identified several mutations that occurred in delta iGluRs of the vertebrate lineage that cause reduced cell surface expression and/or induce inactive, potentially desensitized states. Notably, several of these residues are in the mid-to-rear of the LBD (Fig. 5*D*), a part of the receptor implicated in AMPA/KA iGluR desensitization (40–43), the phenomenon of upper-channel collapse when ligand-bound LBDs separate from each other, loosening the tension between LBDs and channel-forming helices (3). Another vertebrate delta iGluR mutation we identified, G567S in the “hinge” between upper and lower lobes at the rear of the LBD, reduced the GABA selectivity of the invertebrate delta iGluR. How this mutation alters ligand selectivity is not addressed by our experiments, and our dockings to static LBDs do not address, e.g., flexibility of the LBD as determined by residues like G567 in the hinge region. However, previous experimental and computational work on *Rat*GluD2 suggests that D-serine affinity is substantially altered by mutations in the hinge that alter its flexibility (47). That ligand-selectivity may be allosterically controlled is also reflected in the altered glycine/D-serine preference shown by a chimera with an NTD-LBD linker substitution.

Attempting to “reverse” the loss-of-function mutations in vertebrate delta iGluRs and “reawaken” *Rat*GluD2 iGluRs, we found that even when combined, nine reverse substitutions did not reawaken ligand-gated activity. This suggests that additional mutations have accumulated in vertebrate delta iGluRs, and receptor reawakening is now contingent upon these additional residues. Foreseeably, some combination of up to 41 of the potential reverse mutations would achieve this, despite the fact that many of these forward mutations were not noticeably detrimental to starfish delta iGluR function. Reverse mutations in *Rat*GluD2 iGluRs on the *Lc*-mutant background, however, indeed led to ligand-gated channels, either glycine-gated or glycine- and D-serine-gated, depending on the combination of mutations. This confirms previous work showing that delta iGluR LBDs are capable of substantial ligand-induced conformational change (13, 48) and delta iGluR channels are capable of gating (12, 30), but moreover, it shows that only a few amino acid residues prevent the coupling of these two processes in vertebrate delta iGluRs.

Our results also highlight the fact that the effects of upper-M3 mutations are difficult to predict, as they vary depending on amino acids in the LBD. Four of the above reverse mutations in the LBD caused *Rat*GluD2 *Lc*-mutant channels to be closed at rest. This identifies candidate LBD determinants of the differing effects of M3 mutations in different iGluR families (11, 26, 49, 50). Indeed, despite high conservation of upper-M3 amino acid sequence in various delta iGluRs (*SI Appendix*, Fig. S5), we show that the *Lc*-mutation has very different effects in *Rat*GluD2 and *Sac*GluD, as it converts the latter from a generally inactive channel to one that is largely inactive at rest and potently and efficaciously activated by GABA.

Our experiments suggest that mutations in the NTD played relatively little role in the loss of ligand-gated currents in vertebrate delta iGluRs. However, changes in both the NTDs and LBDs may affect delta iGluR activity via a remarkably similar mechanism. Borrowing from broadly accepted mechanisms of AMPA receptor activation and desensitization (4), delta iGluR activity may depend on tight interfaces at the rear of adjacent LBDs so that clamshell closure at the front of the LBD pulls the lower lobe upward, pulling the channel open via the LBD-channel domain linker. Thus, the inter-LBD interface can be stabilized and channel activity enhanced by either introduced disulfides linking NTDs *or* LBDs (19, 37); pre- and intersynaptic proteins that lock delta iGluR NTDs together to keep the receptor taut (17, 19); or amino acid identity in and around the rear of the LBD (present study).

### Pharmacological Avenues.

No selective pharmacological modulators of delta iGluRs are known. Whether such modulators would be directly relevant to pharmacotherapy is unclear, as the links between low GluD1 expression and schizophrenia and GluD2 overactivity and cerebellar ataxia may pertain to early development (51–53) and thus be inaccessible to pharmacotherapy. But delta iGluR modulators would drastically improve physiological experiments aiming to dissect the function of delta iGluRs in neural circuitry in vivo/ex vivo.

Small currents through reawakened vertebrate delta iGluRs and certainly large currents through active invertebrate delta iGluRs offer a tangible experimental system for testing the effects of potential drug molecules on delta iGluR function. Such use of invertebrate receptors would rely on their pharmacological profile matching that of vertebrate GluD1 and GluD2 iGluRs, but our study shows that sensitivity to certain competitive antagonists and pore blockers seems similar in vertebrate and invertebrate delta iGluRs. Our study also suggests that care must be taken when dissecting synaptic iGluR composition with classical AMPA/KA receptor modulators, as some of these also affect delta iGluRs.

### Excitatory GABA Receptors.

The most surprising finding in our study was that many delta iGluRs are activated by the transmitter GABA. GABA mediates most of the rapid *inhibitory* signals in mammalian central synapses via its activation of type-A GABA receptors (GABA_A_ receptors) of the pentameric (or “Cys-loop”) ligand-gated ion channel superfamily (54). The presence of GABA-gated receptors throughout the delta iGluR branch suggests that delta iGluRs became GABA-sensitive early after their emergence, resulting in excitatory GABA receptors that have been inherited by numerous bilaterian animals, including acoels and other xenacoelmorphs, and invertebrate deuterostomes such as starfish and acorn worms. Future studies are needed to determine whether GABAergic neurons synapse onto GABA-gated delta receptors and whether such excitatory GABA receptors also occur in protostomes and early-branching chordates.

There are now numerous examples of ligand-gated ion channels that overturn the conventional view of glutamate as excitatory and GABA and glycine as inhibitory transmitters. Within the iGluR superfamily, there are excitatory glycine-gated NMDA receptors in mammals (55) and numerous glycine-gated and presumably excitatory iGluRs of the “epsilon” family in invertebrates (23, 56). And in the pentameric ligand-gated ion channel superfamily, there are excitatory GABA receptors and inhibitory glutamate receptors (57, 58). The fact that ligand sensitivity and ion permeability are so readily evolvable in different ligand-gated ion channel superfamilies and in different animals means that burgeoning transcriptomic studies must be cautious in assigning neuronal identity to different cells based on the presence of, e.g., iGluR genes. While the prediction of function from sequence will always be susceptible to unidentified switches in function, we foresee that systematic studies of the evolution of receptor families, such as ours, will improve future assessments of neuronal function based on the presence of different ligand-gated ion channel genes.

## Methods

### Phylogenetics.

iGluR amino acid sequences were sought from four chordates (*Rattus norvegicus, Danio rerio, Branchiostoma belcheri,* and *Ciona intestinalis*), two hemichordates *(Ptychodera flava* and *Saccoglossus kowalevskii*), two echinoderms (*Acanthaster planci* and *Anneissia japonica*), one ecdysozoan *(Strigamia maritima*), two spiralians (*Crassostrea gigas* and *Schmidtea mediterranea*), two xenoturbellids (*Xenoturbella profunda* and *Xenoturbella bocki*), two acoels (*Diopisthoporus longitubus* and *Hofstenia miamia*), two nemertodermatids (*Meara stichopi* and *Nemertoderma westbladi*), two cnidarians (*Hydra vulgaris* and *Nematostella vectensis*), one placozoan (*Trichoplax* sp H2), one poriferan (*Oscarella carmela*), and two ctenophores (*Euplokamis dunlapae* and *Pleurobrachia bachei*). Rat sequences were retrieved from UniProt. Other sequences were retrieved via BlastP search with *Rattus norvegicus* GluD2 (NCBI XP_038964327.1) as query in KEGG Genome Database, https://www.genome.jp/kegg/genome/ (*Nematostella vectensis*); Compagen Japan, http://compagen.unit.oist.jp/ (*Osarella carmela* OCAR_T-CDS_130911 dataset); OIST Marine Genomics Unit, https://marinegenomics.oist.jp/acornworm/viewer/info?project_id=33; a published dataset for xenacoelamorphs (59); and NCBI (all other species). We included an additional two plant sequences from *Arabidopsis thaliana* (NCBI) as an outgroup.

Sequences were aligned with MAFFT v7.450 (60) in Geneious Prime (Dotmatics). Sequences that were >95% identical to another and sequences that were excessively long or short were removed. The final alignment contained 246 sequences with 4692 columns (including gaps). From this alignment, we generated a maximum-likelihood phylogenetic tree using IQ-Tree (61) with a Q.pfam+G4 substitution model (62) (log-likelihood of model -410679) and both SH-aLRT (63) and ultrafast bootstrap (64) branch support.

### Molecular Biology and Heterologous Expression.

Delta iGluR sequences were commercially synthesized and subcloned (Genscript Biotech Netherlands) into a custom vector so that finally each coding sequence had silent mutations to remove internal restriction sites, a C-terminal glycine-serine linker and cMyc tag (except for *Saccoglossus kowalevskii* GluD) and was preceded by a loosely Kozak consensus sequence, flanked by *Xenopus laevis* β-globin 5′ and 3′ untranslated sequences, and followed by a poly(A) tail. *Dan*GluD2 and *Cra*GluD sequences were codon-optimized for *Xenopus leavis* in iCodon as we learnt of this software (65). Full delta iGluR sequences in *SI Appendix*, *Supporting Text*. *Rattus norvegicus* GluA2 (flip isoform) in the pRK5 vector was a gift from David MacLean (University of Rochester Medical Center), and human TARPγ2 (CACNG2) in the pcDNA3.1(+) vector was a gift from Stephan Pless (University of Copenhagen). Single- and double-mutant cDNAs were generated by PCR with partly overlapping mutant primers (66) and Phusion Hotstart II High Fidelity polymerase (Fisher Scientific). Chimeras were generated by combining fragments cloned with custom primers using and according to the GenBuilder Cloning Kit (Genscript). Coding sequences of all plasmids were confirmed by Sanger sequencing (Genewiz). Capped mRNA was transcribed from EcoRI (all delta iGluRs), SalI (*Rat*GluA2), or XbaI (TARPγ2) -digested DNAs with the SP6 (delta iGluRs) or T7 (*Rat*GluA2 and *Hom*TARPγ2) Mmessage Mmachine kits (Fisher Scientific) and then purified (RNA Clean & Concentrator kit, Zymo Research). 4 ng (*Aca*GluD), 40 ng (all other delta iGluRs) or 10 ng (*Rat*GluA2 9 ng + *Hom*TARP γ2 1 ng) RNA in 40 nL was injected (Nanoliter 2010, World Pricision Instruments) into collagenase-treated *Xenopus leavis* stage V/VI oocytes (EcoCyte Bioscience) and oocytes were kept in 50% (in water) Leibovitz’s L-15 medium (Gibco) supplemented with additional 0.25 mg/mL gentamicin, 1 mM L-glutamine, and 15 mM HEPES, pH 7.6 at 18 °C for two (*Rat*GluA2) or three to four (delta iGluRs) days before experiments.

### Electrophysiology.

Oocytes were continuously perfused with a bath solution containing (in mM) 96 NaCl, 2 KCl, 1.8 CaCl_2_, 1 MgCl_2_, and 5 HEPES, pH 7.6 with NaOH, in a plastic recording chamber (RC-3Z, Warner Instruments). For zero-Ca^2+^ experiments, CaCl_2_ and MgCl_2_ were replaced with 2 mM BaCl_2_. Oocytes were impaled with Borosilicate micropipettes containing KCl (3 M) and voltage clamped with an OC-725D amplifier (Warner Instruments) controlled via an Axon Digitata interface and pClamp v11 (Molecular Devices). Current was sampled at 1,000 Hz and filtered at 200 Hz. Drugs dissolved in bath solution were applied via an eight-channel, gravity-driven perfusion system (VCS-8-pinch, Warner Instruments). For concanavalin A treatment, oocytes were incubated in dishes of concanavalin A-containing (10 µM) bath solution on ice for 5 to 10 min immediately before recording.

### Immunolabeling.

Oocytes were washed twice with phosphate-buffered saline (PBS) and fixed with 4% paraformaldehyde in PBS overnight at 4 °C. Fixed oocytes were embedded in 3% low-gelling point agarose and sliced with a vibratome in 100-µm sections. Slices were washed with PBS containing 0.2% bovine serum albumin (BSA) and 0.1% Tween 20 for 3 h at room temperature. Slices were incubated with mouse anti-Myc tag monoclonal IgG1 antibody (Myc.A7, Invitrogen MA121316, Fisher Scientific) 1:500 in blocking buffer (PBS containing 1% BSA and 0.1% Tween-20) overnight at 4 °C, washed three times with PBS, incubated with goat anti-mouse polyclonal IgG (H+L) Alexa Fluor 568 conjugate (Invitrogen A11004, Thermofisher) 1:1,000 in blocking buffer for 1 h at room temperature, and washed three times with PBS. Slices were mounted on a glass slide in 50% glycerol, and images were acquired on an Olympus FLUOVIEW FV3000 confocal laser scanning microscope with standard PMT detectors, 20× air and 30× and 40× silicon oil immersion lenses. Images were processed with Fiji software (67).

### Data Analysis.

Currents were additionally filtered (50 Hz, Bessel 8-pole) and measured in Clampfit (Molecular Devices). Data were plotted in Prism v9 (GraphPad). For simple plots, we have shown all data points, but for concentration–response and IV plots, we have shown only mean ± SEM for clarity. EC_50_ values for each individual oocyte were calculated by variable slope nonlinear regression in Prism v9 and used to calculate the means reported in text. Summary concentration–response curves are fit to mean data points in figures.

### Structural Model.

An AlphaFold2 structural model of the *Aca*GluD LBD was created using ColabFold v2 without template information (68, 69). For most figures, this was visualized with PyMol v4.6 (Schrödinger).

### Computational Ligand Docking.

Dockings were conducted using AutoDock 4.2 (70). The structure file generated by AlphaFold2 was used for the docking experiments of *Aca*GluD, while *Rat*GluD2 was docked using the PDB structure file (PDB ID 2v3u) (13) after removing unnecessary molecules such as water and D-serine from the X-ray structure. Both GABA and D-serine molecules used as ligands were retrieved from https://pubchem.ncbi.nlm.nih.gov/ (Compound CID: 119 and 71077 respectively). Employing a blind docking approach, 100 experiments were carried out for each ligand, with a maximum of 25 million energy evaluations per experiment using the Lamarckian genetic algorithm and default parameters from AutoDock4.2. The resulting configurations were clustered ligand binding modes with <2.0 Å rmsd from each other. Clusters of energetically favorable configurations typically included over 10 similar binding modes: Fig. 2 shows the five most energetically favorable; *SI Appendix*, Fig. S3 shows the overall clustering. Images of the ligand-receptor complex were generated using ChimeraX 1.4 (71).

### N-Linked Glycosylation Site Prediction.

The identification of potential N-linked glycosylation sites in *Aca*GluD, *Rat*GluD2, and *Rat*GluD1 was performed using the NetNGlyc 1.0 tool (72) using amino acid sequences as input. The parameters used for the NetNGlyc 1.0 analysis were kept at default settings, and predicted sites were annotated on amino acid sequence (*SI Appendix*, Fig. S5).

## Supplementary Material

Appendix 01 (PDF)

## Data Availability

All study data are included in the article and/or *SI Appendix*.
