# Supplementary material for: Loss of activation by GABA in vertebrate delta ionotropic glutamate receptors
Source: Proc Natl Acad Sci U S A. 2024 Jan 29;121(6):e2313853121. doi: 10.1073/pnas.2313853121 (PMC10861852; doi:10.1073/pnas.2313853121)
Supplement: Supplementary file 1 — Appendix 01 (PDF) [file pnas.2313853121.sapp.pdf]

SUPPORTING INFORMATION FOR:

Loss of activation by GABA in vertebrate delta ionotropic glutamate receptors

Giulio Rosano[a], Allan Barzasi[a], Timothy Lynagh[a,*]

[a] Michael Sars Centre, University of Bergen, 5008 Bergen, Norway

* Correspondence
tim.lynagh@uib.no
Michael Sars Centre, University of Bergen
Thomøhlensgate 55
5008 Bergen
Norway

This PDF file includes:	
Figure S1	Page 2
Figure S2	Page 3
Figure S3	Page 4
Figure S4	Page 5
Figure S5	Page 6
Figure S6	Page 7
Supporting text	Page 8
Supporting information references	Page 12

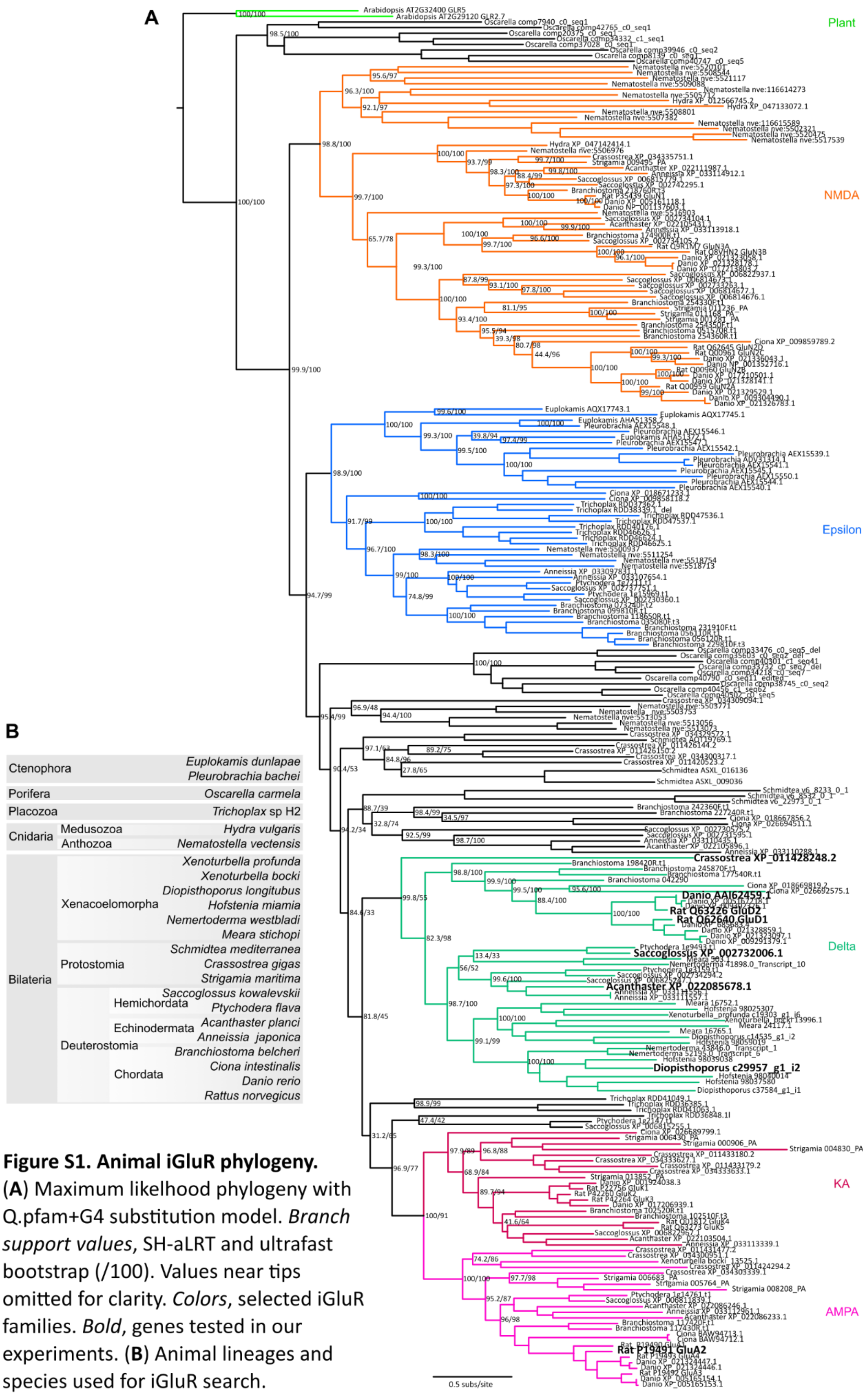

B

Ctenophora			*Euplokamis dunlapae*
			Pleurobrachia bachei
Porifera			*Oscarella carmela*
Placozoa			*Trichoplax* sp H2
Cnidaria	Medusozoa		*Hydra vulgaris*
	Anthozoa		*Nematostella vectensis*
Bilateria	Xenacoelomorpha		*Xenoturbella profunda*
			Xenoturbella bocki
			Diopisthoporus longitubus
			Hofstenia miamia
			Nemertoderma westbladi
			Meara stichopi
	Protostomia		*Schmidtea mediterranea*
			Crassostrea gigas
			Strigamia maritima
	Deuterostomia	Hemichordata	*Saccoglossus kowalevskii*
			Ptychodera flava
		Echinodermata	*Acanthaster planci*
			Anneissia japonica
		Chordata	*Branchiostoma belcheri*
			Ciona intestinalis
			Danio rerio
			Rattus norvegicus

Figure S1. Animal iGluR phylogeny. (**A**) Maximum likelihood phylogeny with Q.pfam+G4 substitution model. *Branch support values*, SH-aLRT and ultrafast bootstrap (/100). Values near tips omitted for clarity. *Colors*, selected iGluR families. *Bold*, genes tested in our experiments. (**B**) Animal lineages and species used for iGluR search.

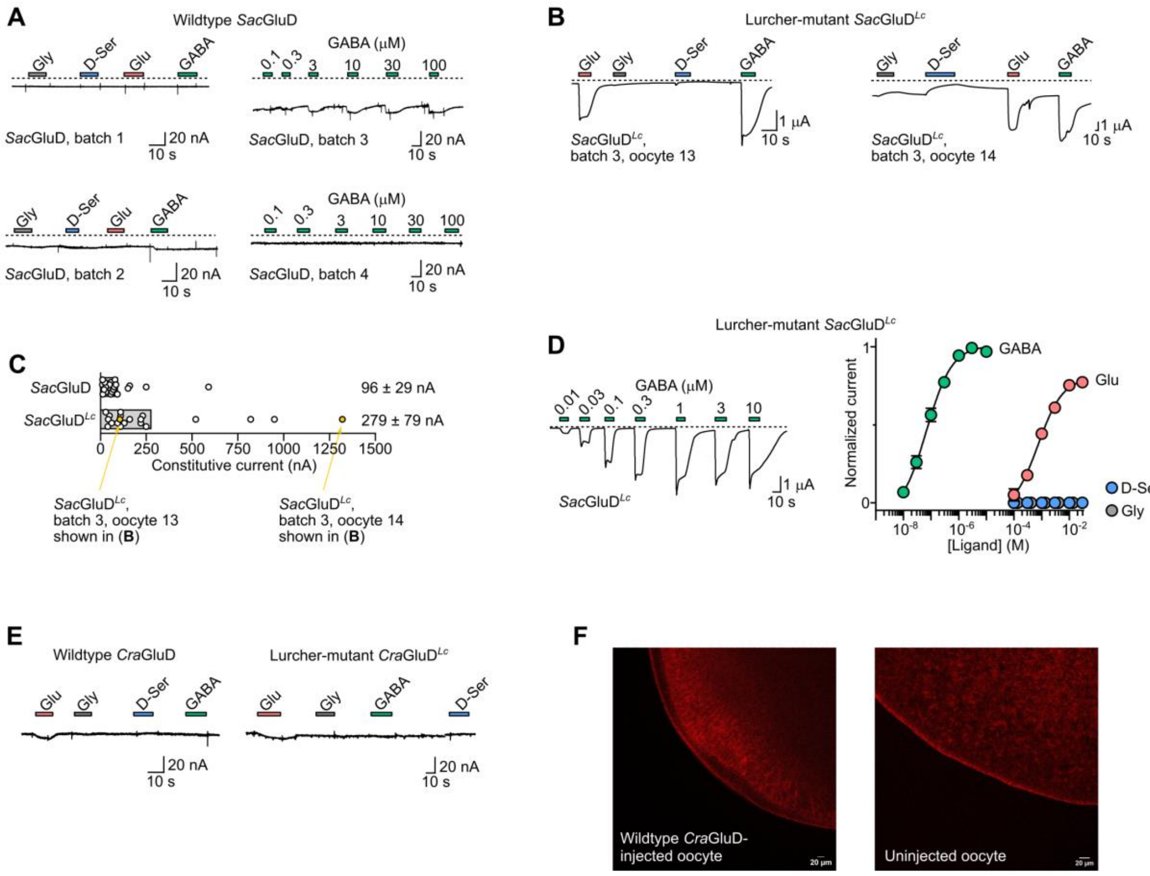

Figure S2. Characterization of *Saccoglossus kowalevskii* and *Crassostrea gigas* GluD iGluRs

(**A**) Two-electrode voltage clamp (TEVC) recordings in oocytes of four different batches, injected with wildtype *Saccoglossus kowalevskii* GluD (*Sac*GluD) mRNA, showing current responses to different potential ligands. *Dashed lines*, zero current.

(**B**) TEVC recordings of responses to different ligands in oocytes injected with lurcher-mutant *Sac*GluD^{Lc}. Oocyte batch number is separate from that in panel **A**. *Dashed lines*, zero current.

(**C**) Mean (bars) and individual data points (both n = 20 oocytes, over four different batches) of constitutive current in wildtype *Sac*GluD-expressing and lurcher-mutant *Sac*GluD^{Lc}-expressing oocytes. Two *Sac*GluD^{Lc} data points are highlighted: these are shown in panel **B**.

(**D**) *Left*, Representative recording, and *right*, mean ± SEM current responses (normalized to GABA-gated current amplitude) to increasing ligand concentrations in oocytes injected with lurcher-mutant *Sac*GluD^{Lc}. n = 4 (GABA), 3 (Glu), or 5 (D-Ser and Gly).

(**E**) Recordings in oocytes injected with wildtype or lurcher-mutant *Crassostrea gigas* GluD (*Cra*GluD or *Cra*GluD^{Lc}) mRNA.

(**F**) Anti-myc fluorescent immunolabelling of oocyte injected with *Cra*GluD-myc mRNA and uninjected oocyte suggests absence of *Cra*GluD surface expression.

A

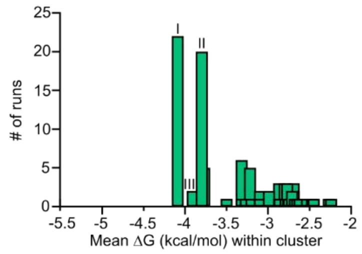

*Aca*GluD model - GABA

Cluster	Rank	ΔG (kcal/mol)	Cluster RMSD (Å)
I	1	-4.53	0
I	2	-4.53	0.16
I	3	-4.45	0.49
II	1	-4.14	0
II	2	-4	1.67
II	3	-3.65	1.27
III	1	-4.07	0
III	2	-3.72	1.87
-	-	-	-

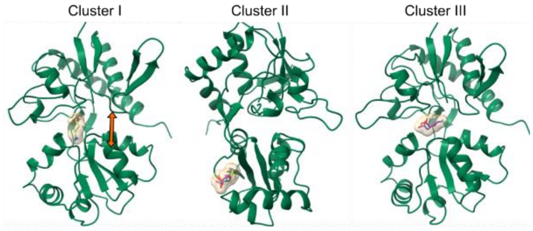

B

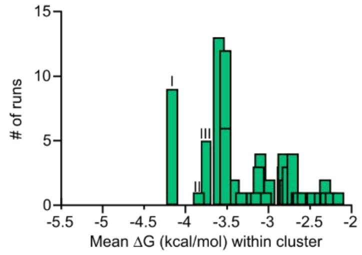

*Aca*GluD model - D-serine

Cluster	Rank	ΔG (kcal/mol)	Cluster RMSD (Å)
I	1	-4.33	0
I	2	-4.32	0.09
I	3	-4.31	0.24
II	1	-3.89	0
II	2	-3.81	0.47
II	3	-3.74	1.74
III	1	-3.86	0
III	2	-3.83	0.23
III	3	-3.81	0.27

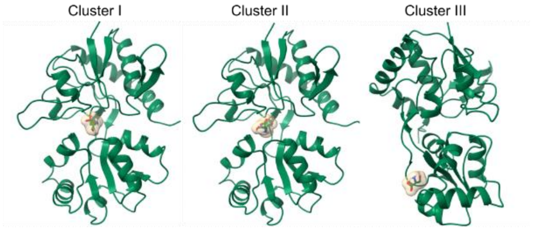

C

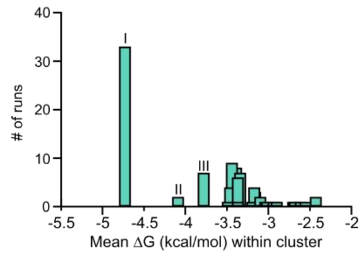

*Rat*GluD2 PDB 2v3u - GABA

Cluster	Rank	ΔG (kcal/mol)	Cluster RMSD (Å)
I	1	-5.57	0
I	2	-5.50	1.02
I	3	-5.48	0.51
II	1	-4.18	0
II	2	-3.99	1.07
-	-	-	-
III	1	-4.12	0
III	2	-4.11	0.17
III	3	-4.04	0.56

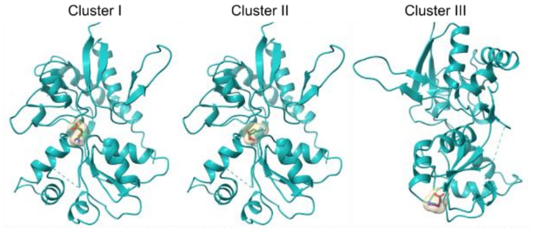

D

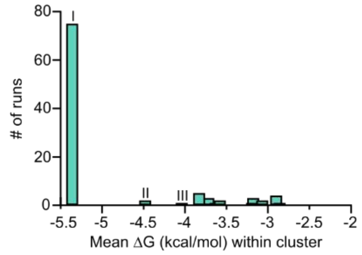

*Rat*GluD2 PDB 2v3u - D-serine

Cluster	Rank	ΔG (kcal/mol)	Cluster RMSD (Å)
I	1	-5.78	0
I	2	-5.75	1.17
I	3	-5.73	0.2
II	1	-4.87	0
II	2	-4.1	0.61
-	-	-	-
III	1	-4.04	0
-	-	-	-
-	-	-	-

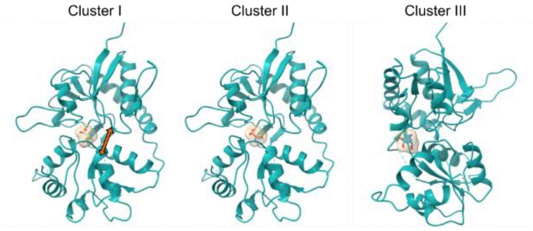

Figure S3. Computational ligand docking to *Aca*GluD and *Rat*GluD2.

(**A**-**D**) Analysis of dockings into clusters of ligand binding modes with <2 Å root mean squared deviation ("RMSD") from each other, for GABA and D-serine at our *Aca*GluD model (**A**,**B**) and the *Rat*GluD2/D-serine structure (PDB 2v3u) with D-serine removed (**C**,**D**). *Left*, number of runs yielding binding modes within clusters (clusters I-III indicated) of decreasing mean binding energy ("mean ΔG within cluster"). *Middle*, corresponding table showing the most favourable binding poses ("Rank 1-3") for the top three clusters. *Right*, graphical representation indicating ligand binding poses in top three clusters. Orange arrows in (**A**) and (**D**) represent *Aca*GluD E475-A747 (**A**) and *Rat*GluD2 E450-A727 (**B**) Cα-Cα distances of 12.4 Å and 12.0 Å.

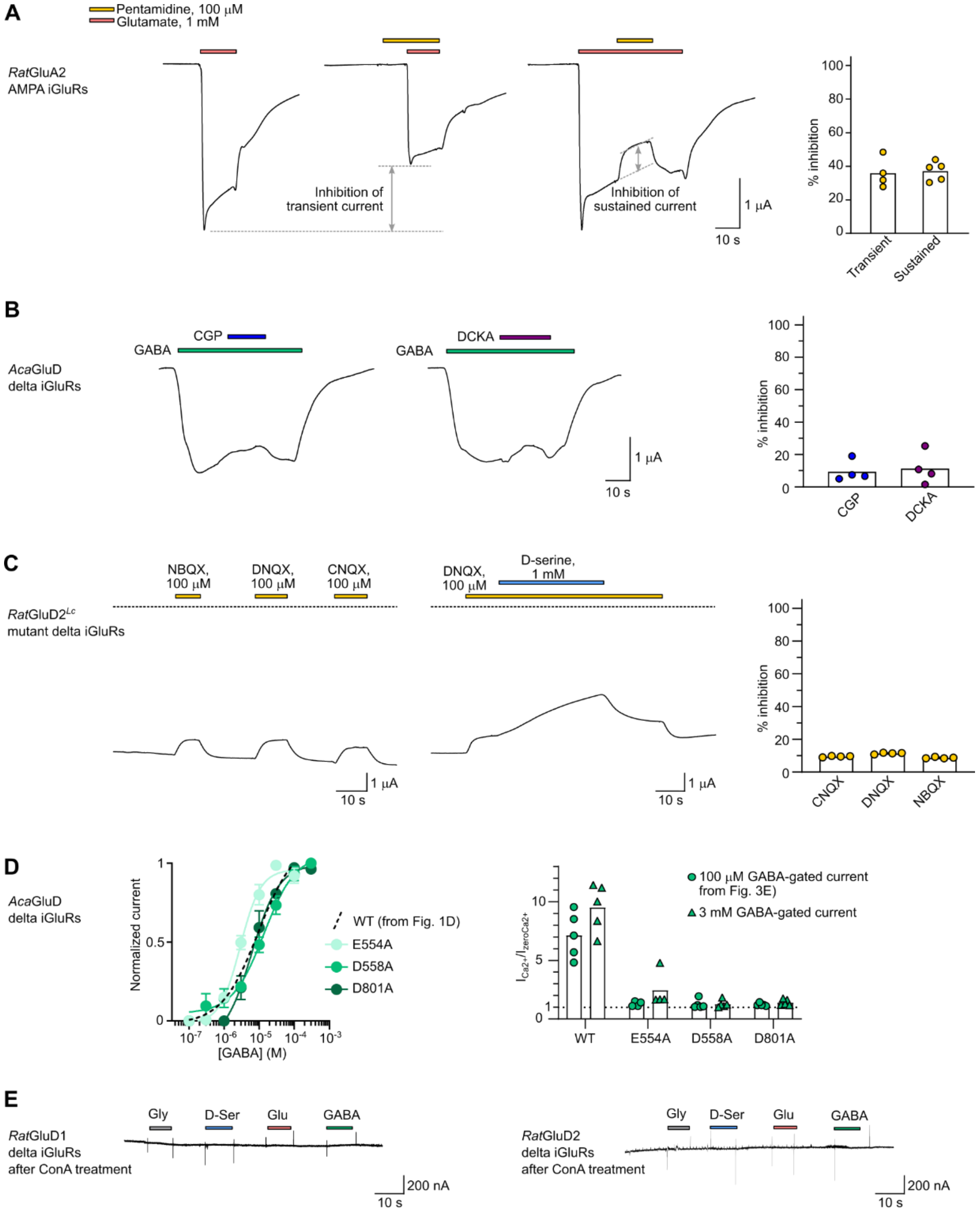

Figure S4. Pharmacological characterization of delta iGluRs and AMPA iGluRs

(**A**) *Left panels*, example recording of glutamate-gated current in an oocyte expressing *Rat*GluA2 and human TARPγ2 (CACNG2). *Right panel*, % inhibition of glutamate-gated current by pentamidine at different oocytes (dots) and resulting means (columns).

(**B**) *Left panels*, example recording of GABA-gated current in an oocyte expressing *Aca*GluD. *Right panel*, % inhibition of GABA-gated current at different oocytes (dots) and resulting means (columns).

(**C**) *Left panels*, example recordings of oocytes expressing A654T lurcher-mutant *Rat*GluD2 (*Rat*GluD2^{Lc}) in response to indicated AMPA iGluR antagonists and D-serine. *Dashed line*, zero current baseline. *Right panel*, % inhibition at different oocytes (dots) and resulting means (columns).

(**D**) *Left*, mean ± SEM (n = 3, D801A; n = 4, all others) normalized (to maximum GABA-gated current at each oocyte) current amplitude in response to increasing GABA concentrations. *Right*, Ca^{2+}-induced enhancement of GABA-gated currents ($I_{Ca2+}/I_{zeroCa2+}$) was similar for 100 μM GABA- and 3 mM GABA-gated currents.

(**E**) Example recordings of oocytes expressing wildtype *Rat*GluD1 or *Rat*GluD2 after oocytes were incubated in 10 μM concanavalin A for 5-10 min.

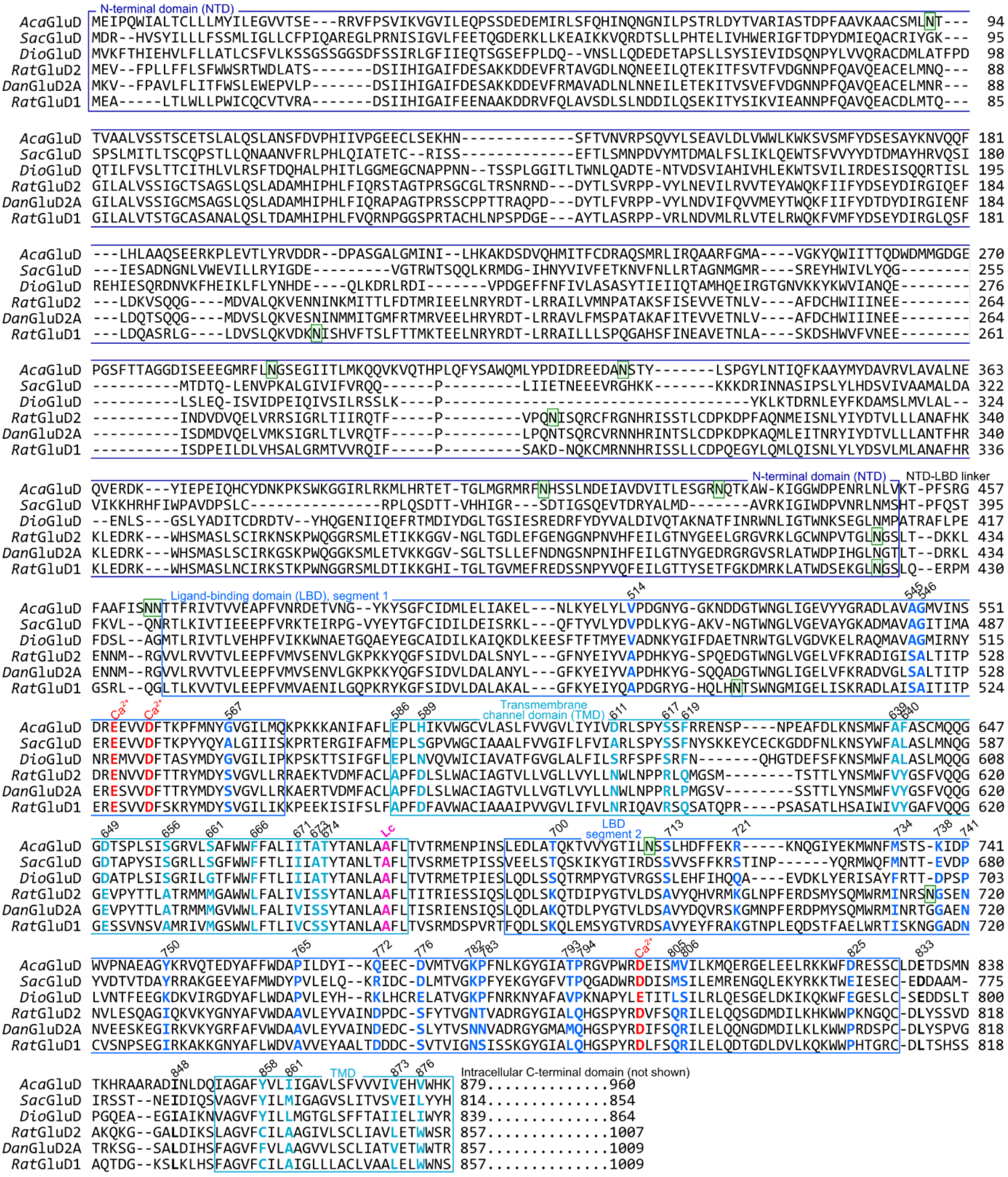

Figure S5. Alignment of active and inactive delta iGluRs

Major tertiary structural elements are indicated as boxes and labeled, as inferred from high-resolution structures of mouse GluD1 N-terminal domain(1) and rat GluD2 ligand-binding domain(2) and moderate-resolution structures of human GluD1 and GluD2 full-length receptors(3, 4). *Bold cyan and blue*, residues that differ between active *Aca*GluD, *Sac*GluD, and *Dio*GluD iGluRs and verified inactive vertebrate delta iGluRs receptors. Numbers refer to *Aca*GluD positions that were mutated. *Pink*, Lurcher mutant (Lc) position(5), mutated to threonine in several Lc mutants in this study. *Red*, potential Ca^{2+}-binding residues(6), mutated to alanine in several mutants in this study. *Green boxes*, predicted N-linked glycosylation sites.

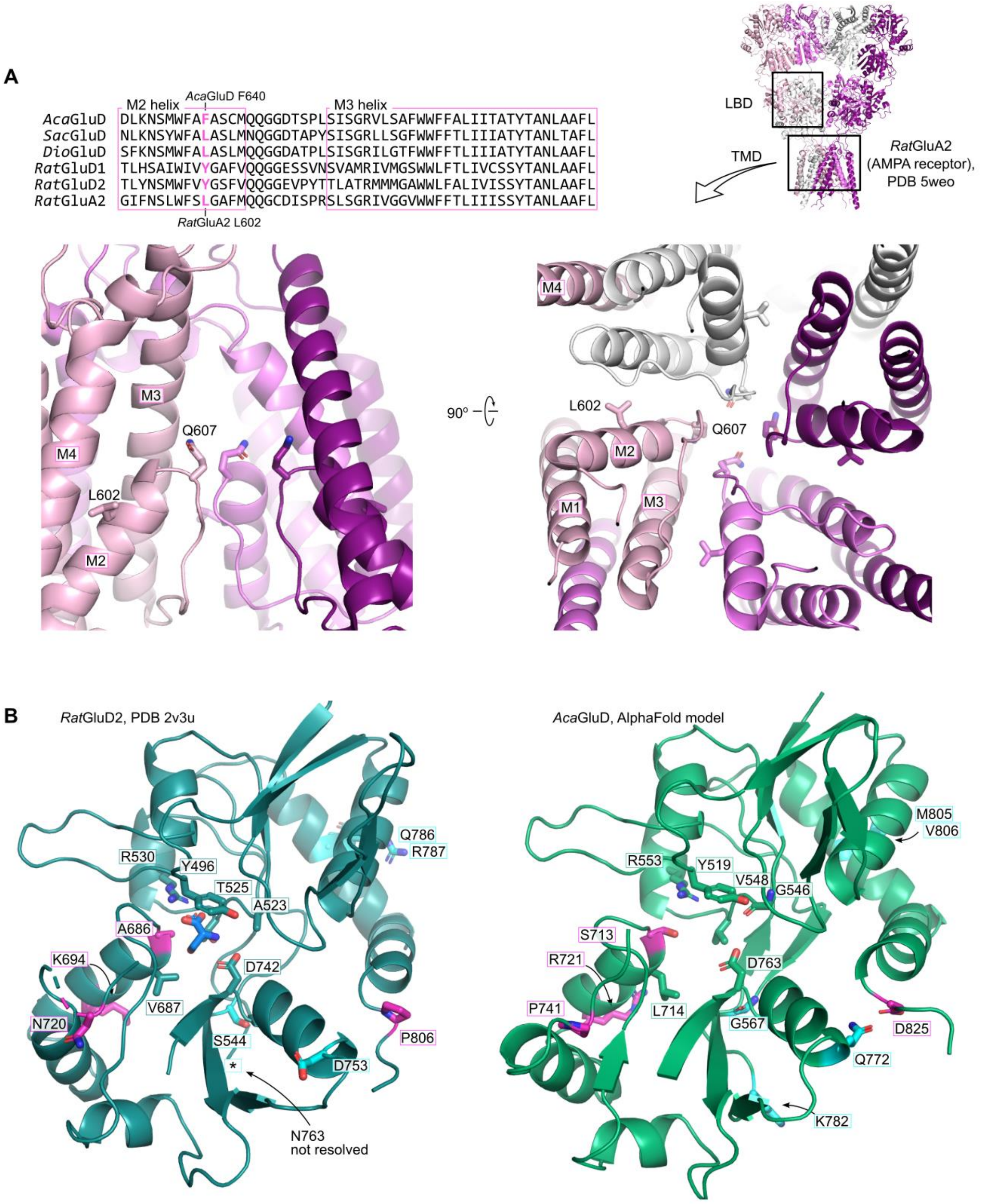

Figure S6. Position of selected amino acid residues in iGluR structures and AlphaFold *Aca*GluD model.
(**A**) Amino acid sequence alignment of membrane-embedded helical segments M2 and M3 from selected delta iGluRs and rat AMPA receptor (*Rat*GluA2, PDB 5weo), and magnified view of the channel pore from cryo-electron microscopy structure of *Rat*GluA2(7), showing the location of *Rat*GluA2 L602, equivalent to *Aca*GluD F640, in comparison to pore-lining residue *Rat*GluA2 Q607. Helices and selected residues for one subunit are labeled. One subunit removed from side view (left) for clarity.
(**B**) *Left*, X-ray structure of D-Serine (blue) -bound *Rat*GluD2 isolated ligand-binding domain (LBD) (2) and *right*, AlphaFold model of *Aca*GluD LBD, highlighting positions whose mutation in *Aca*GluD caused severe (magenta) and moderate (cyan) loss of function and positions that coordinate ligands in most iGluRs (teal in *Rat*GluD2, green in *Aca*GluD). Helix H (residues 723-736 in *Rat*GluD2 and 744-757 in *Aca*GluD) has been hidden from the foreground for clarity.

SUPPORTING TEXT

DNA constructs for expression in *Xenopus laevis* oocytes

All DNA constructs were based on a pSP64 vector (ProMega) modified for expression in *Xenopus* oocytes into which the following delta iGluR inserts were sub-cloned.

```
ATTTAGGTGACACTATAG, SP6 transcription promoter
AAGCTT, HindIII restriction site
GCTTG etc, beta globin 5’ UTR
GTCGACTCTAGAGGATCC, SalI, XbaI, BamHI, restriction sites
ACC, loose Kozak consensus sequence
cMyc + TAA stop codon,
GGTTA etc, beta globin 3’ UTR
AAAAA etc, poly(A) sequence
GAATTC, EcoRI restriction site
```

Modified pSP64 vector

```
ATTTAGGTGACACTATAGAATACAAGCTTGCTTGTTCTTTTTGCAGAAGCTCAGAATAAACGCTCAACTTTGGCGTCGACTCTAGAGGATCCGAGCAGAAGCTCATCAGTGAGGAAGATCTCTAA
GGTTACCACTAAACCAGCCTCAAGAACACCCGAATGGAGTCTCTAAGCTACATAATACCAACTTACACTTTACAAAATGTTGTCCCCCAAAATGTAGCCATTCGTATCTGCTCCTAATAAAAAGA
AAGTTTCTTCACATTCTAAAAAAAAAAAAAAAAAAAAAAAAAAAAAAAACGAATTCGTAATCATGTCATAGCTGTTTCCTGTGTGAAATTGTTATCCGCTCACAATTCCACACAACATACGAGCCGG
AAGCATAAAGTGTAAAGCCTGGGGTGCCTAATGAGTGAGCTAACTCACATTAATTGCGTTGCGCTCACTGCCCGCTTTCCAGTCGGGAAACCTGTCGTGCCAGCTGCATTAATGAATCGGCCAAC
GCGCGGGGAGAGGCGGTTTGCGTATTGGGCGCTCTTCCGCTTCCTCGCTCACTGACTCGCTGCGCTCGGTCGTTCGGCTGCGGCGAGCGGTATCAGCTCACTCAAAGGCGGTAATACGGTTATCC
ACAGAATCAGGGGATAACGCAGGAAAGAACATGTGAGCAAAAGGCCAGCAAAAGGCCAGGAACCGTAAAAAGGCCGCGTTGCTGGCGTTTTTCCATAGGCTCCGCCCCCCTGACGAGCATCACAA
AAATCGACGCTCAAGTCAGAGGTGGCGAAACCCGACAGGACTATAAAGATACCAGGCGTTTCCCCCTGGAAGCTCCCTCGTGCGCTCTCCTGTTCCGACCCTGCCGCTTACCGGATACCTGTCCG
CCTTTCTCCCTTCGGGAAGCGTGGCGCTTTCTCATAGCTCACGCTGTAGGTATCTCAGTTCGGTGTAGGTCGTTCGCTCCAAGCTGGGCTGTGTGCACGAACCCCCCGTTCAGCCCGACCGCTGC
GCCTTATCCGGTAACTATCGTCTTGAGTCCAACCCGGTAAGACACGACTTATCGCCACTGGCAGCAGCCACTGGTAACAGGATTAGCAGAGCGAGGTATGTAGGCGGTGCTACAGAGTTCTTGAA
GTGGTGGCCTAACTACGGCTACACTAGAAGAACAGTATTTGGTATCTGCGCTCTGCTGAAGCCAGTTACCTTCGGAAAAAGAGTTGGTAGCTCTTGATCCGGCAAACAAACCACCGCTGGTAGCG
GTGGTTTTTTTGTTTGCAAGCAGCAGATTACGCGCAGAAAAAAAGGATCTCAAGAAGATCCTTTGATCTTTTCTACGGGGTCTGACGCTCAGTGGAACGAAAACTCACGTTAAGGGATTTTGGTC
ATGAGATTATCAAAAAGGATCTTCACCTAGATCCTTTTAAATTAAAAATGAAGTTTTAAATCAATCTAAAGTATATATGAGTAAACTTGGTCTGACAGTTACCAATGCTTAATCAGTGAGGCACC
TATCTCAGCGATCTGTCTATTTCGTTCATCCATAGTTGCCTGACTCCCCGTCGTGTAGATAACTACGATACGGGAGGGCTTACCATCTGGCCCCAGTGCTGCAATGATACCGCGAGACCCACGCT
CACCGGCTCCAGATTTATCAGCAATAAACCAGCCAGCCGGAAGGGCCGAGCGCAGAAGTGGTCCTGCAACTTTATCCGCCTCCATCCAGTCTATTAATTGTTGCCGGGAAGCTAGAGTAAGTAGT
TCGCCAGTTAATAGTTTGCGCAACGTTGTTGCCATTGCTACAGGCATCGTGGTGTCACGCTCGTCGTTTGGTATGGCTTCATTCAGCTCCGGTTCCCAACGATCAAGGCGAGTTACATGATCCCC
CATGTTGTGCAAAAAAGCGGTTAGCTCCTTCGGTCCTCCGATCGTTGTCAGAAGTAAGTTGGCCGCAGTGTTATCACTCATGGTTATGGCAGCACTGCATAATTCTCTTACTGTCATGCCATCCG
TAAGATGCTTTTCTGTGACTGGTGAGTACTCAACCAAGTCATTCTGAGAATAGTGTATGCGGCGACCGAGTTGCTCTTGCCCGGCGTCAATACGGGATAATACCGCGCCACATAGCAGAACTTTA
AAAGTGCTCATCATTGGAAAACGTTCTTCGGGGCGAAAACTCTCAAGGATCTTACCGCTGTTGAGATCCAGTTCGATGTAACCCACTCGTGCACCCAACTGATCTTCAGCATCTTTTACTTTCAC
CAGCGTTTCTGGGTGAGCAAAAACAGGAAGGCAAAATGCCGCAAAAAAGGGAATAAGGGCGACACGGAAATGTTGAATACTCATACTCTTCCTTTTTCAATATTATTGAAGCATTTATCAGGGTT
ATTGTCTCATGAGCGGATACATATTTGAATGTATTTAGAAAAATAAACAAATAGGGGTTCCGCGCACATTTCCCCGAAAAGTGCCACCTGACGTCTAAGAAACCATTATTATCATGACATTAACC
TATAAAAATAGGCGTATCACGAGGCCCTTTCGTCTCGCGCGTTTCGGTGATGACGGTGAAAACCTCTGACACATGCAGCTCCCGGAGACGGTCACAGCTTGTCTGTAAGCGGATGCCGGGAGCAG
ACAAGCCCGTCAGGGCGCGTCAGCGGGTGTTGGCGGGTGTCGGGGCTGGCTTAACTATGCGGCATCAGAGCAGATTGTACTGAGAGTGCACCATTCGACGCTCTCCCTTATGCGACTCCTGCATT
AGGAAGCAGCCCAGTAGTAGGTTGAGGCCGTTGAGCACCGCCGCCGCAAGGAATGGTGCATGCAAGGAGATGGCGCCCAACAGTCCCCCGGCCACGGGGCCTGCCACCATACCCACGCCGAAACA
AGCGCTCATGAGCCCGAAGTGGCGAGCCCGATCTTCCCCATCGGTGATGTCGGCGATATAGGCGCCAGCAACCGCACCTGTGGCGCCGGTGATGCCGGCCACGATGCGTCCGGCGTAGAGGATCT
GGCTAGCGATGACCCTGCTGATTGGTTCGCTGACCATTTCCGGGTGCGGGACGGCGTTACCAGAAACTCAGAAGGTTCGTCCAACCAAACCGACTCTGACGGCAGTTTACGAGAGAGATGATAGG
GTCTGCTTCAGTAAGCCAGATGCTACACAATTAGGCTTGTACATATTGTCGTTAGAACGCGGCTACAATTAATACATAACCTTATGTATCATACACATACG
```

*Aca*GluD (*Acanthaster planci* delta iGluR, after XM_022229986.1 from Genbank)

```
GTCGACACCATGGAAATACCACAGTGGATAGCTTTAACCTGTCTCCTCCTCATGTATATTCTGGAAGGCGTTGTCACATCTGAGAGGCGTGTTTTCCCGTCAGTCATAAAAGTTGGTGTAATATT
AGAACAGCCGTCGTCAGATGAGGACGAGATGATTCGACTCTCATTCCAACATATAAACCAAAACGGGAACATCTTACCGTCAACCCGATTGGATTATACCGTGGCAAGGATAGCCTCCACAGATC
CCTTTGCAGCCGTTAAAGCAGCATGCTCAATGTTAAATACAACTGTCGCTGCACTTGTGTCTTCTACAAGCTGCGAGACGAGCCTAGCTCTCCAGTCGCTGGCGAACTCTTTCGATGTGCCCCAC
ATTATTGTGCCCGGCGAGGAATGCCTCTCGGAAAAGCATAACAGTTTTACGGTTAACGTGCGACCAAGTCAAGTCTACCTGAGCGAAGCGGTTCTGGACTTGGTATGGTGGCTGAAATGGAAGAG
TGTCTCTATGTTCTACGATTCAGAGAGCGCCTATAAGAACGTGCAGCAGTTCCTGCATCTGGCGGCGCAATCGGAGGAGCGCAAGCCGCTGGAGGTGACGCTGTACCGGGTGGACGATAGGGACC
CGGCCAGCGGCGCGCTTGGGATGATTAACATCTTGCACAAAGCTAAGGATTCGGACGTCCAACACATGATCACCTTCTGCGACAGAGCCCAGAGCATGAGGCTGATTAGACAGGCTGCACGTTTT
GGGATGGCCGTGGGGAAATACCAATGGATTATTACAACACAGGACTGGGACATGATGGGAGATGGCGAGCCGGGGAGCTTCACGACGGCTGGCGGGGACATCTCTGAGGAAGAGGGCATGAGATT
CCTCAATGGCTCCGAGGGCATCATCACCTTGATGAAGCAACAGGTCAAAGTTCAGACCCACCCTCTGCAGTTCTACAGCGCCTGGCAGATGCTCTACCCGGACATCGACAGGGAAGAGGACGCTA
ACTCCACTTACTTGTCGCCGGGGTATCTCAACACGATCCAGTTCAAAGCAGCCTATATGTACGACGCCGTACGAGTATTGGCAGTTGCCTTGAACGAACAGGTTGAGCGAGACAAGTACATTGAA
CCCGAGATACAGCATTGCTATGACAACAAACCCAAATCATGGAAAGGGGGCATTCGCCTCCGGAAGATGCTGCACAGGACGGAAACAACTGGGCTGATGGGACGGATGCGGTTTAATCACAGTAG
TCTGAATGATGAGATAGCGGTGGATGTCATTACCTTAGAAAGTGGCCGTAACCAGACCAAGGCATGGAAGATTGGCGGCTGGGACCCGGAAAATCGTCTAAATTTGGTCAAAACACCCTTTTCAA
GAGGTTTTGCTGCTTTTATCAGTAACAACACCACATTTCGCATTGTAACCGTTGTGGAGGCGCCGTTCGTTAATCGGGACGAGACAGTGAATGGGTACAAGTACTCGGGGTTTTGCATCGACATG
TTAGAACTAATCGCCAAGGAGTTGAACCTGAAGTATGAGTTGTACCTTGTACCAGATGGAAACTACGGAGGAAAGAACGACGACGGGACGTGGAATGGCCTGATTGGGGAAGTTTATTATGGTAG
GGCGGACCTAGCGGTGGCCGGCATGGTCATCAACTCCGACCGCGAGGAAGTGGTGGACTTCACCAAGCCCTTCATGAACTACGGCGTTGGGATTCTCATGCAAAAGCCCAAGAAGAAGGCCAACA
TCTTCGCCTTCCTGGAGCCGCTCCACATCAAGGTCTGGGGCTGCGTGCTGGCCTCCCTCTTCGTGGTCGGGGTGCTGATCTACATCGTGGACCGGCTCAGCCCTTACAGCAGTTTCCGGCGGGAG
AACAGTCCCAACCCGGAAGCCTTCGACTTGAAGAACAGCATGTGGTTTGCCTTCGCTTCCTGCATGCAACAGGGCGGCGACACCTCCCCGCTGTCCATTTCTGGTCGTGTCCTGAGCGCGTTCTG
GTGGTTCTTCGCCCTCATCATCACCGCTACGTACACCGCCAACCTGGCCGCCTTCCTGACCGTCACCCGCATGGAGAACCCCATCAACTCTCTAGAGGATTTGGCCACGCAGAAAACCGTGGTCT
ACGGCACCATCCTTAACAGCAGCCTGCATGACTTCTTCGAGAAGCGAAAGAATCAGGGTATCTACGAAAAGATGTGGAACTTCATGTCCACCTCCAAGATCGACCCCTGGGTGCCCAACGCCGAG
GCGGGGTACAAGCGCGTCCAGACAGAGGACTACGCCTTCTTTTGGGACGCGCCGATCCTAGACTACATCAAGCAGGAGGAGTGTGACGTCATGACTGTGGGCAAGCCCTTCAACCTGAAGGGATA
CGGCATTGCTACGCCGCGGGGGGTTCCATGGAGAGACGAGATATCAATGGTAATCTTAAAAATGCAAGAGAGGGGCGAACTAGAAGAACTCCGTAAGAAGTGGTTCGACCGGGAATCCAGCTGCC
TGGATGAGACGGACAGCATGAATACGAAACATCGCGCCGCCCGGGCCGACATCAACCTGGACCAGATTGCCGGCGCCTTCTACGTCCTGATCATTGGGGCCGTGTTGTCCTTTGTGGTGGTCATT
GTGGAGCACGTTTGGCACAAGCCGTCCTTCTACAAGAAGCGGGAAAAGGAGACGGGAAGGACCACATTGGACTGGTCAGACAAACTCCCTCCACGGGAAACGAGAAACAATGGTCTTTCTAACAT
TGAAAATTGTTCTTCCAATAAGCATTTAGTTGTTCAAGAGAATGACTCTTTTGCGTTAACACCTTTGAGACCAAACCCATGGAGCACAACGACAATTCTTTCTGTCCCCCCTGAAGCGACAGAAA
ACTCTTGCGGATCC
```

*Cra*GluD (*Crassostrea gigantea* delta iGluR, after XM_011429946.3 from Genbank)

```
GTCGACACCATGATACAGTCTACTGGATTTATACTGTTTGTCCTAGCTATTCGATCTTCTGCTAACTGGACAGTGGGAGTCATCTACCAGAGTGACAAGTGGACTGATTTGGGTAAACCACTAAA
TAATGGAAAGGTCACTGCCGTTCAGGAATACCTGTTGCATCAAGTCGATAATTCGGACGCTTTTTCAATGTATCAGAAAGTTTCCAACTTTTCCGAGAGTGGTGTGATTGGAATCGTCGCTTTCA
CATCTTGCCCTACTGCTGCAGTTCTTCAGGAGCAGATCGCTTCTACGAACCTTCCACTGATCACCATAGATAATGAAGTCTGTCCACAAACCTATGACGGCCATCATCATCTGTCCGTGCTTCCA
AACTGCCATAGTCAAAATACACAGTTCATCGATATAGTGCTTCACATGAAGTGGGAACATGTCAACGTCATTTACGATTCAACATCTTCTTACTGCGTTCAGGAACTCATCGTGTTACTGACCAA
AAAGAATGTCTCCAGTAATGCCATATTTCTCCATGAAGGACAACATCTGACGCTGGAAATCTTCCCAGAACTATACTTGGAAGAAGTTATCTACGTGGCTACAGATAAGGAACGGACGCTGGGCA
TTGTCTCCGAGGCTATGACACTGGACATGTTCAACCGTTCCTTCTTCTGGGTTCTGAATTTAGACCCATACGCTCTGTGGAATAGCTCCTTGTATAAGCCTGTCCATGAAGGATGTGTTCTTCTG
ATATCCTCTGGACTTCAACAGAACGAAACCACGGCAGCTGATTTGCTTTCGGAAGCCCTATCAATATTCCAGCGTGGATTAGATTCTATCGGAGATGGACGAAATATCACAGACTTAACATTTCT
TCGCTTTAACCTCAAGCAATACATGATGGGATCTTCCGACGAAACCGGATTAATAGGAAAGGAATCGCAGATCCGGGTTTTCAGTTCTACCAAAGATTACGAAACTCCGTTTGAAGAAGTTGCAG
GTTACTCACAGGATACTCGTAATTTTAATGTAGCTGACGATCGACTTTACAAGTGCATTTTCAAAGACTTTGGCAATAAGACGCTGAAGGTCGTTTCCGTCGAGGGAAAACCTTTCCAAATGCGA
GTGGATAAAGGCAATGGGTCCTTCTACTATTACGGATTTACATACGACCTGCTGAATGAGTTCGCCAAGCAGTTTAACTTCAGATACGAGTTCCTAGATTCAGTAGATGGCCTCTATGGAATAAC
TACAGATGATACCGAAAGTGCTTCAGGGATGATCGGAATGATCATGAGAGGGGAGGCTGATATTGCTGTCGGTGGATTTACAATAACAGCAGACAGAAGAAAAGTTGTAGACTTTCTCTATCCCT
TCCAAGAAGACGGTATCGGAATTATCATGCGAAAGGTTGATAACGAGGCTAACAAGATGTTTCGGGTCTTCAGTTCGCTGGGAACCGACTCTTGGCTTGCTACCGGCGCAGCTGCTTTTGTTGCT
GCCATAGTGTTGTCAATTATCGTCAAGCTGAGTCCGTTTAGTAAGGGACTTCACAACAAGGTATCTGCTAGCTTTTGGTTGGTTTTCTCCGCTTTTCTCCAACAGGGTGAAGAAAAATCCTCCAA
GTCTGCCCCTGCTCGTCTCGTGATAGGATTCTGGTGGATCTTCACAATTATCGTCATGTCCCTGTATACGGCTAACCTTGCTGCCTTTCTGACAGTATCCCTGGCAAAGGCACCAATAAAAAACT
```

TGGAGGAACTTGCAGCTCAGTCCGTTTACAAGCCATTAATTAAGAATGGTTCCAATCTTCACACCCTATTCGAGAAAGCTGAAGGAGGTATCTATAAGAAGATTTGGGATATGATGAGAGATGTC
CCTAAAATCATCTCTCCCGATGAAGGATATGAGATGATCGTCAAAGGAAATTATGCTTACATGACTGATGAATCGGAAGCCCGTTTCAAAGCTATGAAAGACTGCAAGAATCTAGAAATGGCGGA
TGAAACCTTCCATAAAGCAGAGCTCTCTTTCGTTATACGTAAGAACGCAGAGTTTAAAGAGGCTTTCAATAAACACATGCTGAAAATGGTGCAAAATGGCATCGTGGATAAGTACAAGAAGATAT
GGTGGGATAAGCATTCTTGTCATTCGGCTAATACTGCTACTGAACTCGAATTTAAAAGTACAAGTGGAATATTCATTGTCTATTCCGGATTCATATTTATTTCGGTCATCTGCTGTCTTGTAGAG
CTGTACCTTCGAGGTCGTAAATACCCAAAGAACCGTGTCTCATCCAGTAAACAGAGTCTCGAAGATATTTCTGTCATCATGGTTGATTATGGATCC

*Dan*GluD2A (*Danio rerio* delta iGluR, after AAI62459.1from Genbank)

GTCGACACCATGAAAGTTTTTCCGGCAGTACTGTTTCTGATCACATTTTGGTCCCTGGAATGGGAACCTGTTCTTCCCGATTCAATCATTCACATCGGCGCCATTTTTGATGAGTCTGCCAAAAA
GGACGATGAGGTGTTCCGTATGGCAGTCGCGGATCTCAATCTCAACAATGAAATCCTGGAGACAGAGAAGATAACAGTGTCCGTGGAGTTTGTTGATGGAAACAACCCTTTCCAGGCTGTGCAGG
AAGCATGCGAGCTTATGAACAGAGGAATCCTCGCTCTGGTCAGTTCTATCGGCTGCATGTCCGCAGGCTCGCTGCAGTCTTTAGCTGATGCCATGCACATCCCTCACCTGTTCATCCAGAGGGCA
CCCGCGGGCACCCCTCGCTCCAGCTGCCCACCAACAACTCGTGCACAACCCGATGACTACACCCTGTTCGTCCGGCCTCCTGTCTATCTAAATGACGTCATCTTCCAAGTTGTGATGGAGTACAC
TTGGCAGAAGTTCATTATCTTCTACGATACAGACTATGATATCCGTGGAATAGAAAACTTCCTGGACCAGACGTCTCAGCAGGGGATGGATGTGTCTCTGCAGAAGGTTGAATCTAATATTAACA
TGATGATCACTGGAATGTTCCGCACCATGCGAGTGGAAGAACTGCATCGCTACCGAGACACACTGAGACGTGCTGTCCTGTTCATGTCACCCGCCACTGCCAAGGCCTTCATCACCGAAGTGGTG
GAGACCAACCTTGTGGCTTTCGACTGTCACTGGATCATCATAAATGAGGAGATTTCAGATATGGATGTGCAAGAACTGGTCATGAAGTCCATCGGTCGCTTGACCCTGGTGAGACAGACATTCCC
TTTGCCTCAAAACACGAGCCAGCGTTGCGTCCGTAACAATCACCGCATCAACACTTCTCTCTGCGATCCCAAAGACCCTAAGGCACAGATGCTGGAGATTACCAACCGCTACATCTACGACACAG
TACTGTTGCTGGCTAACACATTTCACCGTAAGCTGGAGGATCGTAAGTGGCATTCCATGGCGAGTCTTTCCTGCATCCGTAAGGGCTCCAAACCTTGGCAAGGAGGAAAGTCCATGCTGGAAACT
GTGAAAAAGGGAGGAGTATCTGGTCTGACCTCCCTGCTGGAGTTTAATGATAATGGGTCCAATCCCAACATCCATTTTGAAATCCTGGGTACCAACTACGGTGAGGATCGTGGTCGTGGAGTCTC
CCGTCTGGCTACCTGGGACCCAATCCACGGACTGAATGGAACACTGACCGATAGAAAGCTGGAAAATAACATGCGTGGTGTCGTCCTTCGTGTTGTCACTGTCCTGGAAGAACCGTTTGTTATGG
TGTCTGAAAATGTGCTGGGAAAACCTAAGAAGTACCAGGGATTCTCCATCGATGTGCTGGATGCTCTGGCCAACTATCTCGGATTCAAATATGAGATCTACGTGGCTCCTGATCACAAGTATGGC
AGCCAGCAGGCTGATGGAACGTGGAATGGTCTTATCGGAGAGCTGGTTTTCAAGAGAGCAGACGTAGGACTTTCCGCCCTGACCATCACCCCTGAACGGGAAAGCGTGGTCGATTTCACCACCCG
CTACATGGATTACTCTGTTGGAGTGCTGCTGAGGAAGGCGGAGAGGACCGTCGATATGTTTGCTTGCCTCGCGCCCTTCGACCTGTCCCTGTGGGCTTGTATAGCGGGTACCGTCCTGCTTGTGG
GAACTCTTGTCTATCTGCTCAACTGGCTTAATCCTCCTCGTCTTCCGATGGGCTCTGTCTCCTCAACCACACTCTACAACTCCATGTGGTTTGTCTACGGCTCTTTTGTCCAGCAGGGAGGCGAA
GTGCCCTACACAACTCTGGCTACCCGTATGATGATGGGTGTTTGGTGGCTGTTTGCTCTAATAGTCATCTCCTCCTACACGGCTAATCTGGCTGCTTTTCTTACCATCTCCCGCATTGAGAACTC
CATTCAATCACTGCAGGATTTGGCTAAGCAGACAGATCTCCCTTATGGCACGGTTCTGGACTCCGCCGTCTACGATCAGGTCCGTTCCAAAGGAATGAACCCTTTCGAACGAGACCCTATGTATT
CGCAGATGTGGCGTATGATCAACCGTACAGGAGGAGCCGAAAACAATGTGGAAGAATCAAAAGAAGGAATCCGGAAGGTGAAGTACGGACGTTTTGCATTCGTGTGGGATGCAGCTGTGCTGGAA
TATGTCGCCATTAATGACGAGGACTGTTCGCTGTACACCGTCTCTAACAATGTTGCTGATAGAGGATACGGCATGGCTATGCAGCATGGCAGTCCTTACAGAGACATTTTCTCTCAGCGTATCCT
GGAGCTTCAGCAGAATGGAGATATGGACATCCTGAAGCTTAAGTGGTGGCCAAGGGACAGTCCGTGCGACCTGTACTCTCCAGTCGGAACCCGTAAGAGTGGCAGCGCCCTGGACATCCACAGTT
TTGCTGGAGTCTTCTTTGTTCTAGCGGCAGGTGTGGTCCTGTCCTGTCTGATTGCCACCGTGGAAACGTGGTGGACACGACGTAAGGGCTCCCGTGTCCCATCGAAGGAAGACGATAAAGAAATC
GACCTGGAACACCTGCACCATCGTGTTAACAGCCTGTGCACTGAAGACGAGAGCCCCCACAAACAGTTCTCCACCTCCTCCATCGACCTGACGCCCCTTGATATGGATTCCCTCCCTGCTGCCCG
TCAGGCTCTCGAGCAGATAAGCGACTTCCGTAACACACACATCACCACCACCACCTTTATACCCGAGCAGATTCAGACTCTCTCACGTTCCCTGTCCGCCAAAGCCGCAGCTGGCTTCGCTTTCG
GGGCTGTACAGGATCACCGGACTGGGCGTCCCTTTCGTCAGCGTGCCCCTAATGGCGGCTTCTTTCGCAGCCCTGTCAAAACAATGTCTTCAATCCCTTATCAGCCCACCCCTGCTCCCAACTTT
AGCTATGGAAACGACCCGGATCGAGGAACTTCCATTGGATCC

*Dio*GluD (*Diopisthoporus longitubus* delta iGluR, after c29957 from transcriptome in Andrikou 2019)

GTCGACACCATGGTCAAGTTTACGCACATCGAACACGTGCTTTTCCTTCTGGCGACGTTATGCTCGTTTGTGCTGAAATCATCGGGTAGCAGTGGTGGAAGTGACTTCAGCTCGATACGGCTCGG
ATTCATCATAGAGCAGACGAGTGGAAGTGAGTTTCCACTTGATCAAGTCAATTCCCTGCTTCAGGATGAGGACGAGACAGCTCCTTCGCTCCTTAGCTATAGCATCGAAGTGATCGACTCTCAGA
ACCCGTACCTAGTAGTGCAGCGTGCGTGCGACATGCTAGCGACGTTCCCCGACCAGACGATCCTATTTGTCAGTCTGACAACGTGCATAACTCACCTGGTGCTACGATCGTTCACTGATCAGCAC
GCGCTGCCACACATTACGCTAGGTGGCATGGAAGGGTGTAATGCGCCGCCCAATAACACGTCCTCTCCATTAGGGGGCATCACGCTTACTTGGAATCTACAGGCCGACACAGAGAATACGGTGGA
TTCAGTGATTGCACACATTGTACACTTGGAAAAATGGACGAGTGTAATATTAATACGAGATGAAAGCATCAGCCAACAGCGGACGATCTCGCTACGGGAGCACATTGAAAGTCAGCGGGATAACG
TCAAATTCCATGAGATCAAACTCTTTCTTTACAATCACGATGAGCAGTTGAAGGACCGACTGCGTGACATCGTACCAGATGGAGAGTTTTTCAACTTCATCGTTCTAGCATCTGCTAGCTACACG
ATTGAAATTATACAGACGGCGATGCATCAAGAAATTAGAGGGACTGGAAACGTCAAGAAGTACAAATGGGTGATAGCTAATCAGGAGTTGAGTCTGGAGCAGATTTCGGTGATCGACCCCGAGAT
TCAGATAGTGTCGATTCTGCGATCATCACTCAAACCGTACAAGCTGAAGACAGACCGAAACTTGGAATACTTCAAGGATGCGATGAGTCTGATGGTTCTTGCACTGGAGAACCTTTCGGGAAGTC
TTTATGCTGATATTACTTGCGACAGAGATACAGTCTATCACCAAGGTGAGAACATCATCCAGGAGTTTAGGACTATGGATATCTACGATGGCCTTACTGGTTCGATCGAATCTCGCGAAGACAGA
TTTTACGACTACGTGGCATTGGACATTGTGCAAACAGCCAAGAATGCGACGTTTATCAATCGCTGGAACCTCATCGGTACGTGGAACAAGAGCGAAGGCCTAAACATGCCCGCGACGCGTGCGTT
TCTTCCCGAGTTCGATTCATTGGCGGGCATGACGCTACGAATTGTCACGCTAGTCGAACATCCATTCGTAATCAAAAAGTGGAATGCCGAAACGGGCCAAGCGGAGTACGAAGGCTGCGCTATCG
ACATTCTGAAGGCCATCCAGAAGGATTTGAAGGAAGAGAGTTTCACCTTCACAATGTACGAGGTGGCAGACAACAAATATGGAATTTTCGACGCTGAAACCAATCGCTGGACAGGCCTCGTCGGG
GATGTGAAAGAGCTGAGAGCGCAGATGGCGGTGGCGGGAATGATACGAAACTACAATCGAGAGATGGTAGTGGACTTCACCGCCTCTTACATGGACTACGGAGTGGGTATTCTCATCCCCAAACC
GTCCAAGACGACGAGCATCTTTGGATTTCTGGAGCCACTCAACGTGCAGGTCTGGATCTGTATCGCCGTGGCAACCTTCGGCGTTGGACTCGCGCTCTTCATCCTCTCCCGGTTCTCGCCCTTCA
GTCGGTTCAATCAACACGGAACCGACGAGTTCAGCTTCAAAAACTCAATGTGGTTTGCGCTCGCTAGCCTCATGCAGCAGGGAGGGGATGCGACGCCGCTCTCAATCTCGGGCAGAATCCTCGGC
ACCTTCTGGTGGTTCTTTACTCTTATTATCATAGCGACGTACACGGCGAATTTGGCGGCTTTCCTGACGGTGACGCGTATGGAGACGCCGATCGAGTCCCTTCAAGACCTGAGTAGCCAGACTCG
GATGCCGTATGGGACGGTGCGCGGCAGTTCGCTCGAACATTTCATACACCAGCAGGCGGAGGTAGACAAACTCTACGAGCGCATCTCAGCCTACTTCAGGACAACCGACCCTTCCCCGCTCGTAA
ACACATTCGAAGAAGGCAAAGATAAAGTCATCAGGGGCGATTACGCATTCCTGTGGGACGCTCCCGTGTTGGAGTACCACCGCAAATTACATTGCAGAGAGCTGGCGACGGTGGGAAAGCCATTT
AACCGCAAAAACTATGCCTTCGCCGTGCCAAAAAATGCGCCGTACCTCGAGACGATCACACTGAGCATCCTCCGTCTACAGGAGTCGGGCGAGCTCGACAAAATCAAGCAGAAGTGGTTCGAGGG
TGAGTCCCTGTGTTCGGAGGACGATTCGCTGACACCCGGTCAGGAGGCTGAGGGAATTGCCATCAAGAACGTCGCCGGTGTCTTTTACATACTGCTGATGGGCACCGGACTATCTTTCTTTACGG
CGATCATCGAGTTGATCTGGTATCGCTATTTCAGAGCGATGGAGGACCTGCGTCCTCCTCTACCCCAAAACAGACAGCGGGCCGAATTGTTGGCTCCTACGGGATCC

*Rat*GluD2 (*Rattus norvegicus* delta 2 iGluR, after NM_024379.2 from Genbank)

GTCGACACCATGGAAGTTTTCCCCTTGCTCTTCTTTCTATCCTTCTGGTGGTCTCGAACCTGGGACCTGGCGACCTCCGATTCCATCATCCACATCGGAGCAATTTTTGATGAATCTGCTAAAAA
AGATGATGAAGTATTCCGCACAGCAGTTGGTGACCTCAACCAGAATGAGGAAATCTTACAGACTGAGAAAATCACATTTTCAGTGACATTTGTGGATGGCAACAACCCTTTTCAAGCTGTTCAAG
AAGCATGTGAACTTATGAACCAGGGCATCTTGGCCTTGGTCAGCTCCATTGGTTGCACATCTGCTGGGTCCCTCCAGTCTTTGGCAGACGCCATGCATATCCCTCACCTCTTCATTCAGCGTTCA
ACAGCTGGGACCCCAAGAAGTGGCTGCGGCCTCACCAGGAGCAACAGAAACGATGACTATACCCTTTCAGTTCGTCCACCTGTCTACTTGAATGAAGTCATCCTAAGAGTAGTCACAGAGTATGC
ATGGCAGAAATTTATTATCTTCTATGATAGTGAATATGATATCCGTGGCATACAGGAATTTTTGGACAAAGTTTCCCAGCAGGGAATGGATGTTGCCCTTCAAAAGGTGGAAAACAACATCAATA
AAATGATCACCACGCTCTTTGACACCATGAGGATAGAGGAGTTGAATCGCTATCGAGACACTCTCAGAAGAGCCATCCTTGTTATGAACCCCGCCACAGCCAAATCCTTCATAAGTGAGGTGGTG
GAGACTAATCTGGTTGCTTTTGACTGTCACTGGATCATCATCAATGAGGAAATAAATGATGTGGATGTTCAGGAACTTGTCAGAAGGTCCATTGGAAGGTTAACAATTATTCGGCAGACATTTCC
AGTCCCCCAGAATATAAGTCAGCGCTGTTTCCGTGGCAACCATCGAATTTCTTCAACACTGTGTGATCCCAAGGACCCCTTCGCACAGAATATGGAGATTTCTAACCTTTACATCTATGACACGG
TGCTTCTGCTTGCAAACGCCTTTCATAAGAAGCTGCAGGACCGGAAGTGGCACAGCATGGCGAGCTTGTCCTGTATCAGGAAAAACTCCAAGCCCTGGCAGGGAGGGCGGTCCATGCTGGAGACC
ATCAAGAAGGGTGGAGTTAATGGATTGACTGGAGATCTAGAATTTGGAGAAAATGGAGGTAACCCCAATGTCCACTTCGAAATCCTTGGAACCAACTATGGAGAAGAACTTGGCAGGGGTGTCCG
TAAACTTGGGTGCTGGAATCCTGTCACAGGTCTGAATGGATCACTGACAGACAAGAAACTGGAGAATAACATGCGAGGTGTGGTTCTACGCGTGGTGACTGTATTGGAGGAGCCTTTTGTAATGG
TCTCTGAAAATGTCTTGGGAAAACCGAAGAAATACCAGGGCTTCTCCATTGATGTTTTAGATGCCCTATCTAACTATCTGGGTTTTAACTATGAAATTTATGTTGCACCGGACCACAAATATGGA
AGCCCACAAGAAGATGGGACATGGAATGGACTAGTAGGAGAACTTGTTTTTAAGAGAGCTGACATCGGGATTTCAGCTTTAACCATCACTCCAGACCGGGAGAATGTTGTGGATTTTACAACACG
CTACATGGACTACTCAGTAGGGGTCCTACTGCGAAGGGCAGAAAAAACAGTGGATATGTTTGCCTGCCTTGCACCATTTGATCTATCTCTGTGGGCCTGCATTGCCGGCACAGTCCTTCTTGTGG
GACTCCTGGTCTACCTCTTGAACTGGCTTAATCCCCCACGGTTGCAAATGGGATCAATGACATCTACTACACTCTATAACTCCATGTGGTTTGTGTACGGGTCCTTTGTACAGCAAGGTGGGGAA
GTACCATACACAACTTTGGCAACCCGAATGATGATGGGGGCTTGGTGGTTATTTGCTCTGATTGTCATCTCATCTTATACAGCAAATCTTGCCGCTTTCCTCACTATCACCCGCATTGAGAGCTC
CATCCAGTCTCTTCAGGATCTGTCCAAGCAAACGGATATCCCTTATGGCACAGTCTTGGATTCCGCAGTATATCAGCATGTTCGCATGAAGGGGCTGAATCCTTTTGAGAGGGACAGCATGTATT
CCCAGATGTGGAGGATGATCAACCGAAGCAATGGCTCAGAGAACAATGTTCTGGAGTCCCAAGCAGGCATTCAAAAGGTAAAATATGGAAACTATGCTTTTGTATGGGATGCAGCTGTGTTGGAA
TATGTGGCCATCAATGACCCCGACTGTTCCTTCTACACTGTTGGAAATACTGTTGCTGACCGGGGGTATGGCATCGCACTGCAGCATGGCAGTCCCTACCGTGATGTCTTTTCACAAAGGATTCT
GGAGCTGCAGCAGAGTGGCGACATGGACATCCTCAAGCACAAATGGTGGCCTAAGAATGGCCAGTGTGACCTGTACTCCTCAGTAGATGCAAAGCAGAAGGGAGGAGCCCTGGACATCAAGAGTC
TGGCAGGCGTGTTCTGCATCCTGGCCGCGGGGATTGTACTCTCCTGCCTCATAGCCGTCCTCGAGACATGGTGGAGTCGGCGGAAGGGCTCCCGAGTCCCATCGAAAGAGGATGACAAGGAAATT
GACCTGGAGCACCTCCATAGACGTGTAAATAGCTTGTGCACAGATGACGACAGCCCCCATAAACAGTTTTCCACCTCGTCAATTGACTTGACCCCTCTGGACATTGACACTTTGCCAACACGACA
AGCCCTGGAGCAGATCAGTGATTTCAGAAACACTCACATCACCACCACCACCTTCATCCCAGAGCAGATCCAGACTCTTAGCCGCACACTCTCAGCAAAAGCTGCCTTTGGCTTCACTTTCGGCA
GTGTGCCCGAGCACCGAACTGGCCCTTTTAGGCACAGGGCCCCTAATGGGGGCTTTTTCAGGAGTCCTATCAAAACAATGTCATCTATTCCTTATCAGCCTACTCCCACTCTGGGGCTCAATCTG
GGCAATGACCCCGACCGAGGCACGTCCATAGGATCC

*Rat*GluD1 (*Rattus norvegicus* delta 1 iGluR, after NM_024378.3 from Genbank)

GTCGACACCATGGAAGCGCTGACGCTGTGGCTTCTTCCCTGGATATGCCAGTGCGTTACGGTGCGGGCCGACTCCATCATCCACATCGGTGCCATCTTCGAGGAGAACGCAGCCAAGGACGACAG
AGTGTTCCAGTTGGCTGTATCAGACTTGAGCCTCAATGATGACATCCTGCAGAGTGAGAAGATCACCTACTCCATCAAGGTCATCGAGGCCAATAATCCGTTCCAGGCGGTGCAGGAAGCCTGTG
ACCTCATGACCCAGGGGATTTTGGCCTTGGTCACGTCCACGGGCTGTGCATCTGCCAACGCCCTGCAGTCCCTCACAGACGCCATGCACATCCCACACCTCTTTGTCCAGCGCAACCCCGGGGGT
TCACCACGTACTGCCTGCCACCTGAACCCTAGCCCCGACGGCGAGGCCTACACACTGGCTTCGAGACCGCCCGTCCGTCTCAATGATGTCATGCTCAGGCTGGTGACAGAACTGCGCTGGCAGAA
GTTCGTCATGTTCTATGACAGCGAGTATGATATCCGTGGGCTCCAAAGCTTTCTGGATCAGGCCTCACGGCTGGGTCTTGACGTCTCTTTACAAAAGGTGGACAAGAACATCAGTCACGTGTTCA
CCAGCCTCTTCACCACCATGAAGACGGAGGAGCTGAATCGCTACCGGGACACACTGCGCCGGGCCATCCTCCTGCTTAGCCCACAGGGGGCGCACTCGTTCATCAACGAGGCTGTGGAGACCAAC

TTGGCTTCCAAGGACAGCCACTGGGTCTTCGTGAATGAGGAAATCAGTGACCCCGAGATCCTGGATCTGGTCCACAGTGCCCTTGGCAGGATGACCGTGGTCCGGCAAATCTTCCCATCTGCAAA
GGACAACCAGAAATGCATGAGGAATAACCACCGCATCTCTTCCCTGCTCTGTGATCCACAGGAAGGCTACCTCCAAATGCTGCAGATCTCCAATCTCTATCTGTACGACAGTGTTCTGATGCTGG
CCAACGCCTTCCACAGGAAGCTGGAAGACCGGAAGTGGCATAGTATGGCAAGCCTTAACTGCATACGGAAATCTACCAAGCCATGGAATGGAGGGAGATCCATGCTAGACACCATTAAAAAGGGA
CACATCACCGGCCTCACAGGAGTTATGGAGTTTCGGGAAGACAGCTCAAATCCCTATGTCCAGTTTGAAATCCTTGGCACAACCTATAGTGAGACTTTTGGCAAAGACATGCGCAAGCTGGCGAC
CTGGGACTCAGAGAAGGGCCTGAATGGCAGTCTGCAGGAGAGACCCATGGGCAGCCGCCTCCAAGGACTGACTCTCAAAGTGGTGACTGTCTTGGAAGAGCCTTTTGTGATGGTAGCTGAGAATA
TCCTTGGACAGCCCAAGCGTTACAAAGGGTTCTCCATAGATGTGCTGGATGCATTGGCTAAAGCTCTCGGATTCAAATACGAGATATACCAGGCCCCTGATGGCAGGTATGGCCACCAACTCCAT
AACACTTCCTGGAACGGGATGATCGGGGAGCTCATTAGCAAGAGAGCAGACTTGGCCATCTCTGCTATTACCATCACCCCGGAGAGAGAGAGCGTTGTGGACTTCAGCAAGCGATACATGGACTA
CTCAGTGGGGATTCTCATCAAGAAGCCGGAGGAGAAAATCAGCATCTTCTCCCTTTTCGCCCCCTTTGACTTTGCGGTGTGGGCCTGCATTGCTGCAGCCATTCCCGTGGTGGGTGTGCTCATAT
TCGTGTTGAATCGGATACAGGCTGTAAGGTCTCAGAGTGCCACCCAGCCTCGGCCCTCAGCTTCTGCGACTTTGCACAGTGCCATCTGGATCGTCTATGGAGCCTTTGTCCAGCAAGGTGGTGAG
TCTTCGGTGAACTCTGTGGCCATGCGCATCGTGATGGGCAGCTGGTGGCTCTTCACGCTCATTGTATGTTCCTCCTACACAGCCAACCTTGCTGCTTTCCTCACAGTGTCCAGGATGGACAGCCC
CGTAAGAACATTTCAGGACCTGTCCAAGCAACTGGAGATGTCTTATGGCACTGTCCGGGACTCTGCTGTCTATGAGTACTTCAGAGCCAAGGGGACCAATCCCCTGGAGCAGGATAGCACTTTTG
CTGAGCTCTGGCGGACCATAAGCAAGAATGGAGGGGCTGACAACTGTGTGTCCAATCCTTCAGAAGGTATCAGGAAGGCAAAGAAGGGGAACTACGCCTTTCTGTGGGATGTGGCCGTGGTTGAG
TATGCAGCCCTGACAGATGACGACTGCTCAGTGACTGTCATCGGCAACAGCATCAGCAGCAAGGGCTATGGATTGCCCTGCAACATGGCAGCCCCTACAGGGACCTCTTCTCCCAGAGGATTCT
GGAGCTGCAAGACACAGGGGACCTGGATGTGCTCAAGCAGAAGTGGTGGCCACACACAGGCCGCTGCGACCTCACCAGCCATTCCAGTGCACAGACTGATGGTAAATCCCTTAAGCTGCACAGCT
TCGCTGGGGTCTTCTGCATTTTGGCCATTGGCCTCCTTCTCGCCTGCCTCGTGGCTGCTCTAGAGTTATGGTGGAACAGCAACCGGTGCCACCAGGAGACCCCCAAAGAGGACAAAGAAGTGAAT
CTGGAACAGGTGCACCGGCGCATCAACAGCCTCATGGATGAAGACATTGCTCACAAGCAAATTTCCCCAGCATCCATTGAGCTTTCTGCCCTGGAGATGGGGGGCCTGGCTCCAAGCCAAGCTTT
GGAGCCCACGAGGGAGTACCAGAACACCCAGCTCTCAGTCAGCACCTTTCTGCCTGAGCAGAGCAGCCATGGCACCAGCCGGACACTGTCGTCAGGGCCCAGCAGCAACCTGCCACTGCCGCTGA
GCAGCTCAGCCACCATGCCCTCAATTCAGTGCAAACACAGGTCGCCCAATGGGGGACTGTTTCGACAGAGTCCGGTGAAGACCCCCATCCCTATGTCTTTCCAGCCCGTGCCTGGAGGCGTCCTT
CCAGAGGCCCTGGACACCTCTCATGGCACCTCCATCGGATCC

*Sac*GluD (*Saccoglossus kowalevskii* delta iGluR, after XM_002731960.1 from Genbank)

GTCGACACCATGGATCGACACGTGTCGTATATTCTGTTGTTGTTTTCGAGTATGTTGATAGGCTTGTTGTGTTTTCCGATTCAAGCAAGAGAAGGACTACCTCGCAACATCAGCATAGGTGTGTT
ATTCGAAGAAACTCAAGGAGATGAGAGAAAACTTTTAAAAGAAGCAATCAAAAAAGTTCAAAGGGATACTTCATTATTACCTCACACAGAATTAATTGTTCACTGGGAAAGGATTGGATTCACAG
ATCCGTACGACATGATTGAACAAGCATGCAGAATATATGGGAAGTCGCCTTCATTAATGATAACATTGACGTCCTGCCAGCCATCTACCTTACTGCAAAATGCTGCTAACGTGTTCAGGTTACCA
CATTTACAGATTGCTACGGAGACCTGCAGGATTAGTTCAGAATTTACGCTAAGTATGAATCCCGATGTCTATATGACTGACATGGCGCTGTTTTCGCTGATCAAGTTACAGGAATGGACTTCATT
TGTTGTTTACTATGACACCGATATGGCATACCACCGAGTGCAGAGCATTATTGAATCGGCCGATAATGGTAATCTAGTTTGGGAAGTCATTTTACTACGCTACATTGGCGATGAAGTGGGTACAC
GCTGGACATCACAACAACTCAAAAGAATGGACGGAATACATAATTATGTGATTGTATTTGAAACCAAAAATGTATTTAATCTTTTACGTACGGCAGGTAACATGGGTATGCGGTCACGTGAATAT
CACTGGATCGTTCTTTATCAGGGAATGACAGACACCCAGCTGGAAAATGTGCCTAAGGCATTGGGTATTGTCATATTTGTCAGACAACAACCTCTCATCATAGAAACGAACGAAGAGGAGGTGAG
AGGTCACAAAAAGAAGAAGAAAGATAGAATAAATAATGCAAGTATACCGTCCCTCTATCTCCATGATAGCGTCATTGTTGCTGCCATGGCACTAGATGCAGTTATCAAGAAACACAGGCATTTTA
TTTGGCCCGCAGTAGACCCGTCGTTGTGTAGACCATTACAGTCTGATACTACTGTACATCATATCGGCAGGTCTGATACTATAGGGTCTCAGGAGGTAACGGATAGATATGCGCTAATGGATGCT
GTGCGAAAGATTGGCATATGGGACCCGGTCAACAGGCTGAACATGTCACACACCCCATTTCAGTCCACTTTTAAAGTTCTACAGAACAGGACTTTGAAGATTGTTACTATAGAGGAAGAGCCTTT
TGTTAGAAAAACCGAGATCCGCCCAGGAGTGTATGAATACACAGGTTTCTGCATAGACATATTAGACGAGATTTCCAGAAAGTTACAATTCACGTATGTGCTATATGATGTTCCTGATCTGAAAT
ACGGAGCTAAAGTGAATGGTACATGGAATGGGTTAGTCGGAGAAGTGGCATATGGGAAAGCAGACATGGCAGTAGCTGGAATAACAATAATGGCTGAGCGAGAAGAAGTTGTTGATTTCACTAAA
CCGTATTATCAGTATGCCCTGGGAATTATTATCAGTAAGCCCCGGACCGAGCGAGGCATCTTCGCCTTTATGGAACCATTAAGCGGTCCGGTGTGGGGATGTATAGCAGCAGCCTTGTTTGTAGT
GGGAATATTCCTGTTCGTCATAGCCAGGCTTAGTCCATATAGCTCGTTCAATTACTCTAAAAAAGAATATTGTGAATGTAAAGGCGATGACTTTAACTTGAAGAACAGCTACTGGTTTGCATTGG
CATCCCTTATGAACCAAGGTGGTGATACTGCTCCCTACTCCATCTCCGGGAGATTACTGAGCGGTTTCTGGTGGTTTTTCACTTTAATAATCATAGCTACGTACACAGCTAATTTGACAGCATTT
CTGACAGTGAGCAGGATGGAAACGCCTATATCGTCTGTGGAGGAATTATCAACGCAATCTAAAATCAAATACGGTACAATCCGAGACAGTAGTGTAGTCTCATTTTTTAAAAGATCCACAATAAA
TCCTTATCAGCGAATGTGGCAGTTCATGAATACGACAGAGGTTGATCCATACGTAGACACTGTCACGGACGCATATCGTAGAGCGAAAGGGGAGGAGTATGCCTTCATGTGGGATTATCCCGTGT
TGGAGTTACAAAAAAGAATCGATTGCGATCTTATGACTGTGGGAAAGCCTTTCTATGAAAAGGGATACGGTTTTGTAACTCCACAAGGAGCGGATTGGAGAGATGATATATCCATGAGTATTCTG
GAGATGAGGGAGAATGGTCAACTAGAAAAATATCGCAAGAAGACCTGGGAAATCGAAAGCGAGTGTGAGGACGATGCGGCTATGATAAGAAGTTCCACAAATGAGATAGATATTCAAAGTGTTGC
TGGTGTGTTCTATATACTTATGATCGGTGCAGGTGTCTCCTTGATAACTGTCTCAGTAGAGATATTGTATTATCATTTTTTGAGGAAGTGTTGCCGACCAACTATAACAGTCCATCCCAACGTGG
ATGGTAAGGCTATTGAGGTCAGAGAAGAAAATGCTATAGAAGATGGTAAATCCTTGTCTAATTCCAGAGTTTGAGGATCC

Chimera *Aca*GluD^{RatNTD} (*Acanthaster planci* delta iGluR with NTD replaced with *Rat*GluD2 NTD (purple). *Aca*GluD NTD-LBD linker underlined.)

GTCGACACCATGGAAATACCACAGTGGATAGCTTTAACCTGTCTCCTCCTCATGTATATTCTGGAAGGCGTTGTCACATCTGAGAGGCGTGTTTTCCCGTCCATCATCCACATCGGAGCAATTTT
TGATGAATCTGCTAAAAAAGATGATGAAGTATTCCGCACAGCAGTTGGTGACCTCAACCAGAATGAGGAAATCTTACAGACTGAGAAAATCACATTTTCAGTGACATTTGTGGATGGCAACAACC
CTTTTCAAGCTGTTCAAGAAGCATGTGAACTTATGAACCAGGGCATCTTGGCCTTGGTCAGCTCCATTGGTTGCACATCTGCTGGGTCCCTCCAGTCTTTGGCAGACGCCATGCATATCCCTCAC
CTCTTCATTCAGCGTTCAACAGCTGGGACCCCAAGAAGTGGCTGCGGCCTCACCAGGAGCAACAGAAACGATGACTATACCCTTTCAGTTCGTCCACCTGTCTACTTGAATGAAGTCATCCTAAG
AGTAGTCACAGAGTATGCATGGCAGAAATTTATTATCTTCTATGATAGTGAATATGATATCCGTGGCATACAGGAATTTTTGGACAAAGTTTCCCAGCAGGGAATGGATGTTGCCCTTCAAAAGG
TGGAAAACAACATCAATAAAATGATCACCACGCTCTTTGACACCATGAGGATAGAGGAGTTGAATCGCTATCGAGACACTCTCAGAAGAGCCATCCTTGTTATGAACCCCGCCACAGCCAAATCC
TTCATAAGTGAGGTGGTGGAGACTAATCTGGTTGCTTTTGACTGTCACTGGATCATCATCAATGAGGAAATAAATGATGTGGATGTTCAGGAACTTGTCAGAAGGTCCATTGGAAGGTTAACAAT
TATTCGGCAGACATTTCCAGTCCCCCAGAATATAAGTCAGCGCTGTTTCCGTGGCAACCATCGAATTTCTTCAACACTGTGTGATCCCAAGGACCCCTTCGCACAGAATATGGAGATTTCTAACC
TTTACATCTATGACACGGTGCTTCTGCTTGCAAACGCCTTTCATAAGAAGCTGCAGGACCGGAAGTGGCACAGCATGGCGAGCTTGTCCTGTATCAGGAAAAACTCCAAGCCCTGGCAGGGAGGG
CGGTCCATGCTGGAGACCATCAAGAAGGGTGGAGTTAATGGATTGACTGGAGATCTAGAATTTGGAGAAAATGGAGGTAACCCCAATGTCCACTTCGAAATCCTTGGAACCAACTATGGAGAAGA
ACTTGGCAGGGGTGTCCGTAAACTTGGGTGCTGGAATCCTGTCACAGGTCTGAATGGATCACTGCCCTTTTCAAGAGGTTTTGCTGCTTTTATCAGTAACAACACCACATTTCGCATTGTAACCG
TTGTGGAGGCGCCGTTCGTTAATCGGGACGAGACAGTGAATGGGTACAAGTACTCGGGGTTTTGCATCGACATGTTAGAACTAATCGCCAAGGAGTTGAACCTGAAGTATGAGTTGTACCTTGTA
CCAGATGGAAACTACGGAGGAAAGAACGACGACGGGACGTGGAATGGCCTGATTGGGGAAGTTTATTATGGTAGGGCGGACCTAGCGGTGGCCGGCATGGTCATCAACTCCGACCGCGAGGAAGT
GGTGGACTTCACCAAGCCCTTCATGAACTACGGCGTTGGGATTCTCATGCAAAAGCCCAAGAAGAAGGCCAACATCTTCGCCTTCCTGGAGCCGCTCCACATCAAGGTCTGGGGCTGCGTGCTGG
CCTCCCTCTTCGTGGTCGGGGTGCTGATCTACATCGTGGACCGGCTCAGCCCTTACAGCAGTTTCCGGCGGGAGAACAGTCCCAACCCGGAAGCCTTCGACTTGAAGAACAGCATGTGGTTTGCC
TTCGCTTCCTGCATGCAACAGGGCGGCGACACCTCCCCGCTGTCCATTTCTGGTCGTGTCCTGAGCGCGTTCTGGTGGTTCTTCGCCCTCATCATCACCGCTACGTACACCGCCAACCTGGCCGC
CTTCCTGACCGTCACCCGCATGGAGAACCCCATCAACTCTCTAGAGGATTTGGCCACGCAGAAAACCGTGGTCTACGGCACCATCCTTAACAGCAGCCTGCATGACTTCTTCGAGAAGCGAAAGA
ATCAGGGTATCTACGAAAAGATGTGGAACTTCATGTCCACCTCCAAGATCGACCCCTGGGTGCCCAACGCCGAGGCGGGGTACAAGCGCGTCCAGACAGAGGACTACGCCTTCTTTTGGGACGCG
CCGATCCTAGACTACATCAAGCAGGAGGAGTGTGACGTCATGACTGTGGGCAAGCCCTTCAACCTGAAGGGATACGGCATTGCTACGCCGCGGGGGGTTCCATGGAGAGACGAGATATCAATGGT
AATCTTAAAAATGCAAGAGAGGGGCGAACTAGAAGAACTCCGTAAGAAGTGGTTCGACCGGGAATCCAGCTGCCTGGATGAGACGGACAGCATGAATACGAAACATCGCGCCGCCCGGGCCGACA
sTCAACCTGGACCAGATTGCCGGCGCCTTCTACGTCCTGATCATTGGGGCCGTGTTGTCCTTTGTGGTGGTCATTGTGGAGCACGTTTGGCACAAGCCGTCCTTCTACAAGAAGCGGAAAAGGA
GACGGGAAGGACCACATTGGACTGGTCAGACAAACTCCCTCCACGGGAAACGAGAAACAATGGTCTTTCTAACATTGAAAATTGTTCTTCCAATAAGCATTTAGTTGTTCAAGAGAATGACTCTT
TTGCGTTAACACCTTTGAGACCAAACCCATGGAGCACAACGACAATTCTTTCTGTCCCCCCTGAAGCGACAGAAAACTCTTGCGGATCC

Chimera *Aca*GluD^{RatNTDlink} (*Acanthaster planci* delta iGluR with NTD and NTD-LBD linker replaced with *Rat*GluD2 NTD and NTD-LBD linker (purple). *Rat*GluD2 NTD-LBD linker underlined.)

GTCGACACCATGGAAATACCACAGTGGATAGCTTTAACCTGTCTCCTCCTCATGTATATTCTGGAAGGCGTTGTCACATCTGAGAGGCGTGTTTTCCCGTCCATCATCCACATCGGAGCAATTTT
TGATGAATCTGCTAAAAAAGATGATGAAGTATTCCGCACAGCAGTTGGTGACCTCAACCAGAATGAGGAAATCTTACAGACTGAGAAAATCACATTTTCAGTGACATTTGTGGATGGCAACAACC
CTTTTCAAGCTGTTCAAGAAGCATGTGAACTTATGAACCAGGGCATCTTGGCCTTGGTCAGCTCCATTGGTTGCACATCTGCTGGGTCCCTCCAGTCTTTGGCAGACGCCATGCATATCCCTCAC
CTCTTCATTCAGCGTTCAACAGCTGGGACCCCAAGAAGTGGCTGCGGCCTCACCAGGAGCAACAGAAACGATGACTATACCCTTTCAGTTCGTCCACCTGTCTACTTGAATGAAGTCATCCTAAG
AGTAGTCACAGAGTATGCATGGCAGAAATTTATTATCTTCTATGATAGTGAATATGATATCCGTGGCATACAGGAATTTTTGGACAAAGTTTCCCAGCAGGGAATGGATGTTGCCCTTCAAAAGG
TGGAAAACAACATCAATAAAATGATCACCACGCTCTTTGACACCATGAGGATAGAGGAGTTGAATCGCTATCGAGACACTCTCAGAAGAGCCATCCTTGTTATGAACCCCGCCACAGCCAAATCC
TTCATAAGTGAGGTGGTGGAGACTAATCTGGTTGCTTTTGACTGTCACTGGATCATCATCAATGAGGAAATAAATGATGTGGATGTTCAGGAACTTGTCAGAAGGTCCATTGGAAGGTTAACAAT
TATTCGGCAGACATTTCCAGTCCCCCAGAATATAAGTCAGCGCTGTTTCCGTGGCAACCATCGAATTTCTTCAACACTGTGTGATCCCAAGGACCCCTTCGCACAGAATATGGAGATTTCTAACC
TTTACATCTATGACACGGTGCTTCTGCTTGCAAACGCCTTTCATAAGAAGCTGCAGGACCGGAAGTGGCACAGCATGGCGAGCTTGTCCTGTATCAGGAAAAACTCCAAGCCCTGGCAGGGAGGG
CGGTCCATGCTGGAGACCATCAAGAAGGGTGGAGTTAATGGATTGACTGGAGATCTAGAATTTGGAGAAAATGGAGGTAACCCCAATGTCCACTTCGAAATCCTTGGAACCAACTATGGAGAAGA
ACTTGGCAGGGGTGTCCGTAAACTTGGGTGCTGGAATCCTGTCACAGGTCTGAATGGATCACTGACAGACAAGAAACTGGAGAATAACATGCGAACATTTCGCATTGTAACCGTTGTGGAGGCGC
CGTTCGTTAATCGGGACGAGACAGTGAATGGGTACAAGTACTCGGGGTTTTGCATCGACATGTTAGAACTAATCGCCAAGGAGTTGAACCTGAAGTATGAGTTGTACCTTGTACCAGATGGAAAC
TACGGAGGAAAGAACGACGACGGGACGTGGAATGGCCTGATTGGGGAAGTTTATTATGGTAGGGCGGACCTAGCGGTGGCCGGCATGGTCATCAACTCCGACCGCGAGGAAGTGGTGGACTTCAC
CAAGCCCTTCATGAACTACGGCGTTGGGATTCTCATGCAAAAGCCCAAGAAGAAGGCCAACATCTTCGCCTTCCTGGAGCCGCTCCACATCAAGGTCTGGGGCTGCGTGCTGGCCTCCCTCTTCG
TGGTCGGGGTGCTGATCTACATCGTGGACCGGCTCAGCCCTTACAGCAGTTTCCGGCGGGAGAACAGTCCCAACCCGGAAGCCTTCGACTTGAAGAACAGCATGTGGTTTGCCTTCGCTTCCTGC
ATGCAACAGGGCGGCGACACCTCCCCGCTGTCCATTTCTGGTCGTGTCCTGAGCGCGTTCTGGTGGTTCTTCGCCCTCATCATCACCGCTACGTACACCGCCAACCTGGCCGCCTTCCTGACCGT
CACCCGCATGGAGAACCCCATCAACTCTCTAGAGGATTTGGCCACGCAGAAAACCGTGGTCTACGGCACCATCCTTAACAGCAGCCTGCATGACTTCTTCGAGAAGCGAAAGAATCAGGGTATCT
ACGAAAAGATGTGGAACTTCATGTCCACCTCCAAGATCGACCCCTGGGTGCCCAACGCCGAGGCGGGGTACAAGCGCGTCCAGACAGAGGACTACGCCTTCTTTTGGGACGCGCCGATCCTAGAC
TACATCAAGCAGGAGGAGTGTGACGTCATGACTGTGGGCAAGCCCTTCAACCTGAAGGGATACGGCATTGCTACGCCGCGGGGGGTTCCATGGAGAGACGAGATATCAATGGTAATCTTAAAAAT
GCAAGAGAGGGGCGAACTAGAAGAACTCCGTAAGAAGTGGTTCGACCGGGAATCCAGCTGCCTGGATGAGACGGACAGCATGAATACGAAACATCGCGCCGCCCGGGCCGACATCAACCTGGACC

AGATTGCCGGCGCCTTCTACGTCCTGATCATTGGGGCCGTGTTGTCCTTTGTGGTGGTCATTGTGGAGCACGTTTGGCACAAGCCGTCCTTCTACAAGAAGCGGGAAAAGGAGACGGGAAGGACC
ACATTGGACTGGTCAGACAAACTCCCTCCACGGGAAACGAGAAACAATGGTCTTTCTAACATTGAAAATTGTTCTTCCAATAAGCATTTAGTTGTTCAAGAGAATGACTCTTTTGCGTTAACACC
TTTGAGACCAAACCCATGGAGCACAACGACAATTCTTTCTGTCCCCCCTGAAGCGACAGAAAACTCTTGCGGATCC

SUPPORTING INFORMATION REFERENCES

1. J. Elegheert *et al.*, Structural basis for integration of GluD receptors within synaptic organizer complexes. *Science* **353**, 295-299 (2016).
2. P. Naur *et al.*, Ionotropic glutamate-like receptor delta2 binds D-serine and glycine. *Proc Natl Acad Sci U S A* **104**, 14116-14121 (2007).
3. A. P. Burada, R. Vinnakota, J. Kumar, The architecture of GluD2 ionotropic delta glutamate receptor elucidated by cryo-EM. *J Struct Biol* **211**, 107546 (2020).
4. A. P. Burada, R. Vinnakota, J. Kumar, Cryo-EM structures of the ionotropic glutamate receptor GluD1 reveal a non-swapped architecture. *Nat Struct Mol Biol* **27**, 84-91 (2020).
5. J. Zuo *et al.*, Neurodegeneration in Lurcher mice caused by mutation in delta2 glutamate receptor gene. *Nature* **388**, 769-773 (1997).
6. K. B. Hansen *et al.*, Modulation of the dimer interface at ionotropic glutamate-like receptor delta2 by D-serine and extracellular calcium. *J Neurosci* **29**, 907-917 (2009).
7. E. C. Twomey, M. V. Yelshanskaya, R. A. Grassucci, J. Frank, A. I. Sobolevsky, Channel opening and gating mechanism in AMPA-subtype glutamate receptors. *Nature* **549**, 60-65 (2017).
